# Novel and Founder Pathogenic Variants in X-Linked Alport Syndrome Families in Greece

**DOI:** 10.3390/genes13122203

**Published:** 2022-11-24

**Authors:** Despina Hadjipanagi, Gregory Papagregoriou, Constantina Koutsofti, Christiana Polydorou, Polichronis Alivanis, Aimilios Andrikos, Stalo Christodoulidou, Manthos Dardamanis, Athanasios A. Diamantopoulos, Anastasios Fountoglou, Eleni Frangou, Eleni Georgaki, Ioannis Giannikouris, Velissarios Gkinis, Pavlos C. Goudas, Rigas G. Kalaitzidis, Nikolaos Kaperonis, Georgios Koutroumpas, George Makrydimas, Grigorios Myserlis, Andromachi Mitsioni, Christos Paliouras, Fotios Papachristou, Dorothea Papadopoulou, Nikolaos Papagalanis, Aikaterini Papagianni, Garyfalia Perysinaki, Ekaterini Siomou, Konstantinos Sombolos, Ioannis Tzanakis, Georgios V. Vergoulas, Nicoletta Printza, Constantinos Deltas

**Affiliations:** 1 biobank.cy Center of Excellence in Biobanking and Biomedical Research, University of Cyprus, Nicosia 2109, Cyprus; 2 Department of Biological Sciences, University of Cyprus, Nicosia 2109, Cyprus; 3 Department of Nephrology, General Hospital of Rhodes, 85133 Rhodes, Greece; 4 Department of Nephrology, “Hatzikosta” General Hospital of Ioannina, 45445 Ioannina, Greece; 5 Department of Nephrology, “Evangelismos” General Hospital of Athens, 10676 Athens, Greece; 6 Department of Nephrology, General Hospital of Preveza, 48100 Preveza, Greece; 7 Department of Nephrology, “St. Andrew” General State Hospital, 26332 Patra, Greece; 8 Nephroxenia Dialysis Unit, 45221 Ioannina, Greece; 9 Department of Nephrology, Limassol General Hospital, SHSO, Limassol 4131, Cyprus; 10 Medical School of the University of Nicosia, Engomi, Nicosia 2408, Cyprus; 11 Pediatric Nephrology Unit, “IASO” Children’s Hospital, 15123 Maroussi, Greece; 12 Department of Nephrology and Hemodialysis Unit, Mediterraneo Hospital, 16675 Glyfada, Greece; 13 Medifil SA Private Nephrological Center, 12132 Peristeri, Greece; 14 Olympion, Dialysis Center, 27200 Amaliada, Greece; 15 Department of Nephrology, University Hospital of Ioannina, 45500 Ioannina, Greece; 16 Department of Nephrology, Hellenic Red Cross Hospital “Korgialeneio-Benakeio”, 11526 Athens, Greece; 17 Hemodialysis Unit, “Achillopouleion” General Hospital of Volos, 38222 Volos, Greece; 18 Department of Obstetrics and Gynecology, University Hospital of Ioannina, School of Health Sciences, Faculty of Medicine, University of Ioannina, 45110 Ioannina, Greece; 19 Organ Transplantation Unit, “Hippokration” General Hospital of Thessaloniki, Aristotle University of Thessaloniki, 54642 Thessaloniki, Greece; 20 Department of Nephrology, “P. & A. Kyriakou” Children’s Hospital, 11527 Athens, Greece; 21 Pediatric Nephrology Unit, 1st Department of Pediatrics, “Hippokration” General Hospital of Thessaloniki, Aristotle University of Thessaloniki, 54643 Thessaloniki, Greece; 22 Department of Nephrology, “Papageorgiou” General Hospital of Thessaloniki, 56429 Thessaloniki, Greece; 23 Department of Nephrology, “Hippokration” General Hospital of Thessaloniki, Aristotle University of Thessaloniki, 54643 Thessaloniki, Greece; 24 Division of Nephrology, General Hospital of Rethymnon, 74100 Rethymno, Greece; 25 Department of Pediatrics, University Hospital of Ioannina, Faculty of Medicine, University of Ioannina, 45500 Ioannina, Greece; 26 Renal Unit, “George Papanikolaou” General Hospital of Thessaloniki, 57010 Thessaloniki, Greece; 27 Department of Nephrology, General Hospital of Chania “Agios Georgios”, 73300 Chania, Greece; 28 School of Medicine, University of Cyprus, Nicosia 2109, Cyprus

**Keywords:** Alport syndrome, glomerular basement membrane, Collagen IV, *COL4A5*, founder mutation, pathogenic variant, next generation sequencing

## Abstract

Alport syndrome (AS) is the most frequent monogenic inherited glomerulopathy and is also genetically and clinically heterogeneous. It is caused by semi-dominant pathogenic variants in the X-linked *COL4A5* (NM_000495.5) gene or recessive variants in the *COL4A3*/*COL4A4* (NM_000091.4/NM_000092.4) genes. The disease manifests in early childhood with persistent microhematuria and can progress to proteinuria and kidney failure in adolescence or early adulthood if left untreated. On biopsy, pathognomonic features include alternate thinning, thickening and lamellation of the glomerular basement membrane (GBM), in the presence of podocyte foot process effacement. Although previous studies indicate a prevalence of AS of about 1/50,000, a recent publication reported a predicted rate of pathogenic *COL4A5* variants of 1/2320. We herewith present 98 patients (40 M/58 F) from 26 Greek families. We are selectively presenting the families segregating the X-linked form of AS with pathogenic variants in the *COL4A5* gene. We found 21 different pathogenic variants, 12 novel: eight glycine and one proline substitutions in the collagenous domain, one cysteine substitution in the NC1 domain, two premature termination of translation codons, three splicing variants, one 5-bp insertion/frameshift variant, one indel-frameshift variant and four gross deletions. Notably, patients in six families we describe here and three families we reported previously, carried the *COL4A5*-p.G624D substitution, a founder defect encountered all over Europe which is hypomorphic with mostly milder symptomatology. Importantly, on several occasions, the correct genetic diagnosis reclassified patients as patients with AS, leading to termination of previous immunosuppressive/cyclosporine A therapy and a switch to angiotensin converting enzyme inhibitors (ACEi). With the understanding that all 98 patients span a wide range of ages from infancy to late adulthood, 15 patients (11 M/4 F) reached kidney failure and 11 (10 M/1 F) received a transplant. The prospects of avoiding lengthy diagnostic investigations and erroneous medications, and the advantage of delaying kidney failure with very early administration of renin-angiotensin-aldosterone system (RAAS) blockade, highlights the importance of timely documentation of AS by genetic diagnosis.

## 1. Introduction

Alport Syndrome (AS) is a genetically heterogeneous inherited glomerulopathy that first manifests in early childhood as persistent microscopic hematuria. If left untreated, the condition progresses to proteinuria and chronic kidney function decline and may reach end-stage renal disease (ESRD) before the age of 30 years [1,2]. The most frequent form of the disease follows X-linked inheritance with pathogenic variants in the *COL4A5* gene, whereas a rarer form is caused by homozygous or compound heterozygous changes in either the *COL4A3* or the *COL4A4* genes, which map head-to-head on chromosome 2q36 and share common regulatory sequences [3,4,5]. Women heterozygous for *COL4A5* pathogenic variants also present with microscopic hematuria and run a high risk of kidney failure, with 15% of them developing ESRD by the age of 60 years [6,7]. Digenic inheritance with co-inherited pathogenic variants in two of the three genes is a rare event and results in variable phenotypes [8].

AS is characterized by the development of extrarenal features such as hearing loss in about 60% of cases and ocular abnormalities in about 44% of the patients [7]. Ultrastructural pathognomonic features include thinning and thickening of the glomerular basement membrane (GBM) which is accompanied by GBM splitting and lamellation and podocyte foot process effacement. Sclerotic features, including focal segmental glomerulosclerosis (FSGS) of renal biopsy (identified using light microscopy), are also common [9,10].

Collagen IV genes are large structures, encoded by an average of 50 exons, with the characteristic (Gly-X-Y)n repetitive sequence, where X and Y positions are frequently occupied by prolines. Therefore, they are highly GC-rich and are frequent targets for mutations, especially substitutions of glycine residues in the central collagenous domain by other bulkier amino acids. Nearly every glycine substitution in the repetitive (Gly-X-Y)n motif leads to a phenotype.

Recent findings from large population databases showed that mutations in any one of the three genes, *COL4A3/A4/A5*, are highly prevalent, with about 1/2000 carrying glycine pathogenic variants in the *COL4A5* gene and 1/106 carrying glycine variants in the *COL4A3/A4* genes. Evidently, such heterozygous defects demonstrate incomplete penetrance [11], as they are not encountered with this frequency in outpatient clinical settings. In addition to the familiar microscopic hematuria, heterozygous mutations in the *COL4A3/A4* present with histological uniform thinning of the GBM, largely known as thin basement membrane nephropathy (TBMN). Perhaps owing to the co-inheritance of multiple genetic modifiers, many people with heterozygous variants are highly predisposed to TBMN and may develop late onset severe symptoms, with some requiring renal replacement therapy at a mean age of 56 years [12,13]. Several authors diagnose these patients with autosomal dominant AS [14,15], although they rarely develop extrarenal symptoms or ultrastructural features pathognomonic of AS [16,17,18]. In a recent publication, a very rare form of TBMN and Alport-like nephropathy, caused by recessive mutations in the *P3H2* gene which encodes the prolyl-3 hydroxylase gene, was reported [19].

In this work we diagnosed 98 patients from 26 Greek families with the X-linked form of AS and identified 21 pathogenic variants, including 12 novel variants in the *COL4A5* gene. In six families, we found the European-wide founder pathogenic variant *COL4A5*-p.G624D, which was previously associated with a milder course of disease and a later age of onset of ESRD [20,21,22]. The age at referral to a genetics lab varied between the different nephrology units in Greece and a renal biopsy was still frequently performed before DNA testing. Documentation of the AS diagnosis as early as possible by DNA testing is a great advantage when considering that early blockade of the renin-angiotensin-aldosterone system (RAAS) with ACE inhibitors effectively delays disease progression and onset of ESRD [23].

## 2. Materials and Methods

### 2.1. Patients and Families

We diagnosed 98 patients from 26 families from Greece for which there was suspicion for AS based on clinical, biochemical and urine findings, or a biopsy result when available. The proband in each family was first examined molecularly and the identified candidate DNA variant was then tested in the rest of the at-risk family members. A prominent feature was the presence of microscopic hematuria of glomerular origin. For many family members, in addition to microscopic hematuria, their symptoms included variable proteinuria and chronic renal failure, as well as severe rapid deterioration of renal function, reaching end-stage renal disease, thus requiring renal replacement therapy or kidney transplantation. Hearing loss was reported in 21 patients (21.4%, 18 M/3 F) and ocular problems were reported in only five males (5.1%). Renal biopsies were performed in 17 patients from 15 families (9 M/8 F), providing additional information concerning pathognomonic histological features of the kidneys such as alternate thinning and thickening of the GBM, even focal segmental glomerulosclerosis, and podocyte foot process effacement. This work was approved by the Cyprus National Bioethics Committee and all samples and data were anonymised before being used in this study, in compliance with the European general data protection regulation (GDPR, (ΕΕ) 2016/679).

### 2.2. Amplicon-Based Next-Generation Sequencing (NGS) and Pathogenicity Variant Assessment

DNA from peripheral blood leucocytes was isolated by a salting out procedure [24] or by using a commercially available column-based kit (Qiagen, Hilden, Germany). The molecular testing procedure included NGS performed on IonTorrent PGM (LifeTechnologies, Los Angeles, CA, USA). We used the Ion Ampliseq Designer platform (V3.0, LifeTechnologies, CA, USA) to design and implement a custom NGS panel of five genes (*COL4A3*, *COL4A4*, *COL4A5*, *CFHR5* and *FN1*) which consisted of 260 amplicons in two primer pools to cover 99.25% of the genes. Amplicon size ranged from 125–275 bp to include exonic regions with ±10 bp padding and covered a total of 48.69 kb. We have previously demonstrated the validity of this panel, using 66 samples from 27 families with heterozygous pathogenic variants in the *COL4A3/A4* genes [18]. Probands from 26 families were processed by NGS in specific batches, ensuring a minimum depth of coverage of 20x. Highly suspicious variants identified in *COL4A5* through NGS were independently validated by Sanger re-sequencing with the ABI PRISM 3130xl genetic analyzer, using relevant primers and the ABI BigDye Terminator v1.1 Cycle Sequencing Kit (ABI3130xl, Applied Biosystems, Foster City, CA, USA) (Table S1).

Following NGS analysis, base calling and alignment against hg19 (GRCh37) genome assembly were performed by IonTorrent PGM. This was done as part of the running protocol to eventually generate BAM files which were subsequently uploaded to the IonReporter v5.18 cloud platform (LifeTechnologies, CA, USA) using previously established parameters [25]. Each previously reported variant was investigated, noting its Minor Allele Frequency (MAF) if available, and by searching in specific databases: in-house database, HGMD v2022.3, LOVD, ARUP ALPORT, Ensembl, ClinVar and dbSNP. Additionally, to determine the potential pathogenic effect of each DNA variant, in silico assessment was performed by utilizing at least five different available algorithms, namely SIFT (deleterious variants have scores <0.05), PolyPhen-2 and Grantham (higher values indicate increased probability of the variant being damaging), Mutation Taster and meta-predictor tool REVEL (pathogenicity probability increases as score values approach 1), and SNPs3D (positive score indicates a deleterious variant) [26,27,28,29,30]. We also considered ACMG classification, based on criteria provided on the Varsome platform, for the interpretation of sequence variants [31] (Table 1). When needed, other methods, such as restriction digest of mutated amplified PCR products (RFLP) which uses primers positioned into the flanking introns and specific restriction enzymes, were also performed as additional means of verification (Table S1). The presence of variants in the family members studied was also examined using the above approaches. If a variant had not been previously reported, its frequency was specifically investigated in 50–100 healthy subjects from the general population (controls). Selected samples were processed by multiplex ligation-dependent probe amplification (MLPA) using commercially available kits to screen for *COL4A5* gross aberrations (SALSA MLPA Probemixes P191 and P192 Alport-mixes, MRC Holland, NL, Amsterdam, The Netherlands).

## 3. Results

We studied clinically and molecularly patients from 26 Greek families. In most cases, there was a family history, while renal biopsy data were available from patients in 15 families.

We used an NGS approach with a panel of five genes, namely *COL4A3*, *COL4A4*, *COL4A5*, *CFHR5* and *FN1*. *FN1* pathogenic variants are a very rare cause of hematuria and proteinuria with fibronectin deposits [59]. *CFHR5* encodes a protein for complement activation regulation. An exon 2–3 deletion is endemic in Cyprus [60]. In this cohort, patient ages ranged from infancy to advanced adulthood. Degree of renal function impairment varied across patients, with some having reached ESRD (Table 2). Clinical details were not available for all.

**Molecular studies and clinical context**: We are presenting data from families where we found pathogenic variants in the *COL4A5* gene. With the exception of patients carrying pathogenic variant *COL4A5*-p.G624D (see below), the patients inheriting other pathogenic variants developed typical X-linked AS if male, or a milder phenotype if female. Candidate DNA variants were first identified in the proband through NGS analysis and then verified by Sanger DNA re-sequencing or MLPA in a diagnostics lab. Altogether, 21 different pathogenic variants were detected, 12 of which were novel. All family pedigrees are shown in Figure S1 (includes updated pedigrees from another three families carrying pathogenic variant *COL4A5*-p.G624D, which were previously reported on). We used PhenoTips (REF https://pubmed.ncbi.nlm.nih.gov/23636887/ accessed on 13 October 2022), which uses the open-source tool “Open-Pedigree”as its pedigree editor for the creation of the pedigrees, to manage family and clinical data.

**Families GR1.21-GR1.26 (Figure S1.21–S1.26) (Table 2 and Table 3):** In six families, a founder pathogenic variant was identified, *COL4A5*-p.G624D, which was previously described in another three Greek families (Table 3, GR1.27*, GR1.28*, GR1.29*) [22,42]. It is a hypomorphic variant of pan-European presence and variable phenotypic severity. In most patients, it causes a milder disease and an age of onset of ESRD about 20–30 years later (mean 54 years old) than the age of onset associated with most other glycine substitutions [21]. In many patients, the phenotype is more reminiscent to that of TBMN rather than typical AS.

Table 3 tabulates data for 52 patients of the nine Greek families (21 M/31 F). Note that out of 21 males, four reached ESRD at the advanced ages of 51, 39, 46 and 61 years. No females progressed to kidney failure. In family GR1.24, in a 42-year-old male, the initial erroneous diagnosis of mesangial proliferative glomerulonephritis/IgA nephropathy was reclassified to AS, resulting in termination of immunosuppressive treatment. Three males, aged 28, 72 and 81 years, were eventually diagnosed correctly and their diagnostic odyssey ended. A 50-year-old male was eventually diagnosed correctly and proceeded to transplantation, while two males at ESRD with unknown aetiology were legitimately diagnosed. In total, only three males developed hearing loss and none had eye abnormalities.

Previously, we attributed the hypomorphic nature and variable expressivity of *COL4A5*-p.G624D to its position near the 12th natural interruption of the *COL4A5* α-chain triple helical domain. It converts the G1G collagenous sequence interruption to a G4G interruption, and may therefore cause less of a disruption to the zipper-like propagation of the triple helix [42].

This is the most prevalent pathogenic variant in the Polish population, accounting for 39% of X-linked AS in unrelated Polish families [21]. It also represents 48% of all *COL4A5* pathogenic variants in a population sequencing database [11]. Zurowska et al. calculated that this DNA variant probably first occurred during the 12th–13th centuries. Based on information from the Genome Aggregation Database (gnomAD database), this variant has a frequency of 1/5000 in the European non-Finnish population [21].

The pathogenicity of the filtered variants was classified using the ACMG criteria plus any other information that facilitated its evaluation. Most variants were classified as Pathogenic.

**Family CR1.1 (Figure S1.1):** DNA variant *COL4A5*-c.2719C > T, p.P907S, is a substitution of proline by serine. Prolines are highly conserved in collagens as they contribute to the triple helix stability and flexibility. ACMG classifies it as Likely Pathogenic. Its pathogenicity is enhanced by the observation that this variant was inherited by a mother who reached ESRD at the young age of 39 years, and by her three daughters who presented microscopic hematuria since infancy. Two brothers of the mother for which we did not have DNA, reached ESRD at 15 and 19 years old and were transplanted at 19 and 26 years old, respectively, while their mother started renal replacement therapy at the age of 58 years. Also, one of her daughters had a biopsy at 20 years old which showed, via electron microscopy analysis, GBM defects pathognomonic for AS with significantly thickened and irregular texture lamina densa, lamellation and podocyte foot processes focally fused in glomeruli.

**Family GR1.11 (Figure S1.11):** *COL4A5*-c.4428C > G, p.C1476W is a substitution of cysteine by tryptophan, in the NC1 domain. This cysteine participates in intrachain disulfide bridge formation with cysteine 1567, thus promoting the correct three-dimensional alpha5 chain structure, which is essential during the collagen inter-chain recognition and subsequent triple helix propagation towards the NH_2_-terminal. This variant not only annuls the disulfide bridge, but it also introduces a much bulkier residue, tryptophan, which presumably interferes with correct chain folding. The variant is classified by ACMG and several other in silico algorithms as Pathogenic. The variant was never found in databases, like gnomAD. It is inherited by three affected members, including the father-proband and his two daughters, while it is not inherited by the proband’s healthy brother. The affected father had reached ESRD and received a transplant at 23 years old, with his heterozygous mother as donor. He is currently being prepared for a second transplant from his wife. His older sister is heterozygous and healthy without clinical details.

**Families GR1.4-GR1.6, GR1.8, GR1.13, GR1.18, GR1.20 (Figures S1.4–S1.6, S1.8, S1.13, S1.18, S1.20):** Each family has its own DNA variant, which is a glycine substitution by a bulkier amino acid. Eighteen members (8 M/10 F) inherited variants classified by ACMG as Likely Pathogenic or Pathogenic. All have a REVEL score close to 1.0 and are classified as Pathogenic. One male reached ESRD before the age of 30 years and one female reached ESRD in her late fifties. We had data for six patients, four males and two females, who developed hearing loss (Table 2). In all cases for which data are available, there is segregation of the phenotype with the variant status. Family GR1.18 refers to a boy with GBM findings pathognomonic of AS and hearing loss at the age of 14 years. Family GR1.20 represents a single female with both thin basement membranes and thickening. She had hematuria at 3 years old and proteinuria since the age of 9 years. In families GR1.18 and GR1.20, none of the tested parents carried the variant, raising the possibility that they were de novo events.

It is well documented that substitutions of glycines of the (Gly-X-Y)n repeat are predisposing to triple helix instability. In a previously published systematic analysis on 70 pathogenic variants, we showed that the bulkier the side chain of the amino acid substituting for the glycine (as reflected in the number of carbon atoms), the earlier the age of onset of ESRD [61,62]. This is because collagen biochemistry dictates that when bulkier amino acids are positioned where the three helical α chains meet when wound around each other, this creates steric hindrance which interferes with the normal ultrastructural triple helix formation.

**Families GR1.15, GR1.16 (Figures S1.15, S1.16):** Both families inherited premature termination codons, in a total of six patients (2 M/4 F). Biopsy images were not pathognomonic, only suggestive for AS, while one female reached ESRD at 54 years old. Two males and one female developed hearing loss.

**Families GR1.2, GR1.7, GR1.17 (Figures S1.2, S1.7, S1.17):** Two families segregated pathogenic variants affecting the canonical splice site consensus and the third one, GR1.7, the +5 G close to the donor in intron 7. Although it is not part of the ±1–2 nucleotides at the canonical splice site donor sequence, which is 100% conserved, this +5 position is evolutionarily partly conserved in many genes, while several previous publications reported on this same variant in patients with AS (Table 2) (see also [63]. In this family, the proband and his uncle (who reached ESRD) are affected. After renal transplantation he developed membranous nephropathy in the graft. His sister has isolated microscopic hematuria.

**Figure 1:**
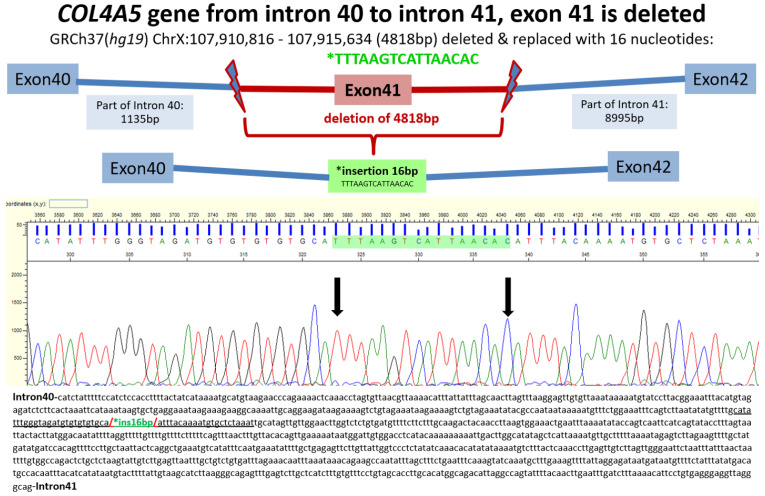
A gross genomic deletion was first detected with MLPA analysis, encompassing exon 41 in the *COL4A5* gene of the proband in family GR1.14. Several attempts with gap PCR using intronic primers flanking exon 41 followed by Sanger sequencing permitted the exact mapping of the deletion. The deletion spanned a sequence of 4818 bp [chrX:107,910,816-107,915,634 in genome assembly GRCh37(hg19)], which was replaced with a 16 bp insertion (TTTAAGTCATTAACAC). The deletion extended from intron 40 to intron 41, including exon 41. Underlined is part of the sequence that is represented in the electropherogram.

**Families GR1.3, GR1.9, GR1.10, GR1.12, GR1.14, GR1.19 (Figures S1.3, S1.9, S1.10, S1.12, S1.14, S1.19):** These families segregate major structural alterations, exon deletions, one 5-bp insertion duplicating CATGG (GR1.12), and one indel (deleting ATA/inserting T), thereby resulting in a reading frameshift and premature termination of translation. Amongst 20 patients (9 M/11 F), eight males developed hearing loss, four in family GR1.3, three in GR1.9 and one in GR1.14. In the same families, six males (ages 17, 19, 24, 25, 27, 36 years) and one female at age 41 reached ESRD. Six males received a transplant. In family GR1.14, we could map the exact borders of exon 41 deletion, extending from intron 40 to intron 41. At the position of the deletion, 16 novel bp were inserted (Figure 1). Although the numbers are small, the relatively higher frequency of hearing loss and ESRD could pertain to the gross mutagenic alterations in these families.

In family GR1.10, a mother and daughter had episodes of macroscopic hematuria after streptococcal infection. The mother sustained two biopsies with suspicion for mesangioproliferative glomerulonephritis/IgA nephropathy. She was on cyclosporine therapy until molecular testing established the *COL4A5* deletion of ivs1-ivs41, after which she and her daughter were clinically reclassified as patients with AS and received ACE inhibitors. In the absence of a previous family history, we suspect this is a de novo change. Although we did not examine this further, we hypothesize that the mother might be mosaic, which explains why her daughter is heterozygous with a more severe hematuria and proteinuria. In family GR1.12, the mother of the proband is negative for the pathogenic variant and the father is reportedly healthy, thereby suggesting that the CATGG duplication is another de novo event.

## 4. Discussion

AS is a clearly monogenic disorder. Therefore, molecular testing promises that AS can be unequivocally diagnosed if a pathogenic DNA variant is detected which means that clinicians can avoid invasive renal biopsies. AS experts suggest that genetic testing be performed in all cases of persistent familial microscopic hematuria. For individuals with persistent proteinuria and steroid-resistant nephrotic syndrome, they also recommend genetic testing for the *COL4A* genes to exclude inherited FSGS and kidney failure due to an unknown cause [64]. Despite the recommendation that genetic testing be used as a first-tier approach, our data indicate that a biopsy is still preferred in many nephrology centers in Greece, as was the case for patients from 15 families. Another explanation might be that there is no easy access to a molecular diagnostics lab with adequate expertise on collagen IV nephropathies, while the co-occurrence of findings could complicate differential diagnosis. To the best of our knowledge, there are currently only two published studies on AS that report genetic findings about the X-linked *COL4A5* gene in the Greek population, both performed in our laboratory [18,42]. Another report described a skin biopsy for the diagnosis of AS in a 3-year old boy [65].

Establishing the genetic diagnosis for AS will provide useful knowledge based on inheritance pattern (autosomal or X-linked) and prognostic information regarding the anticipated age to reach kidney failure, as glycine substitutions are known to confer a lower risk to ESRD compared to frameshift defects or gross structural alterations [7,34]. At the same time, distinguishing AS from any other glomerulopathy may avoid erroneous immunosuppressive regimens and prompt the administration of renoprotective therapies, since several landmark clinical trials have demonstrated the positive outcome of pharmacological interventions targeting the RAAS blockade [66,67,68]. Importantly, recent clinical trials support the use of ACE inhibitors at very early stages of the disease, even when presenting with only low-grade of proteinuria (urine albumin <300 mg/g creatinine or isolated hematuria), for which genetic documentation of an AS diagnosis is needed [23,69]. Other treatments are also under scrutiny, while new ones are being explored [70,71]. SGLT2-mediated correction of the hemodynamic overload of the glomerular filtration barrier appears to be a very promising therapeutic approach [72].

In pursuing precision medicine, genetic testing is the gold standard technique for diagnosing AS; nevertheless, it is not always possible to have a molecular finding that will resolve the diagnostic challenge. Amino acid substitutions for glycine residues in the central collagenous domain are most often pathogenic although there are exceptions that include variants [*COL4A4*-c.1634G > C, p.G545A_rs1800516] and [*COL4A4*-c.2996G > A, p.G999E_ rs13027659] (see ref. [18]). Reportedly, there is some discrepancy between variant annotation using ACMG criteria compared to evaluating variants through scoring algorithms alone. Although the former takes the latter into account, it is frequently challenging to confidently report novel variants identified by NGS based solely on the ACMG criteria [31]. The availability of family segregation data for novel variants can be of great help even when no robust functional studies are available. Convincing family segregation data may permit reclassifying a variant of unknown significance (VUS) to a likely pathogenic or even pathogenic one, thereby establishing the diagnosis and enabling family counselling and programming. An exemplar case in this cohort is family GR1.7 where the *COL4A5*(ivs7)-c.438 + 5G > A variant is classified as a VUS when implementing the ACMG criteria, but classified as “pathogenic” by prediction algorithms (see data on Table 2). The G at the +5 position of introns is highly conserved through evolution, although to a lesser degree than the canonical GT at positions +1 and +2. The variant is shared by three affected family members, and not shared by two at-risk but healthy family members.

Similarly, in five other families where the DNA variant is classified by ACMG as “Likely Pathogenic”, we provide supporting information based on the inheritance in the respective families. These are: GR1.4 (*COL4A5*-p.G669D), CR1.1 (*COL4A5*-p.P907S), GR1.6 (*COL4A5*-p.G908E), GR1.8 (*COL4A5*-p.G1015E), GR1.5 (*COL4A5*-p.G1258S) (Table 1 and Table 2 and Figure S1 with pedigrees). The variants p.G908E and p.G1015E were previously reported in other publications and are included in the HGMD database (Table 1). [HGMD Professional: https://www.hgmd.cf.ac.uk/ac/index.php accessed on 13 October 2022]

Here, we report on 21 pathogenic variants explaining AS in 26 X-linked families with 98 carriers of pathogenic or likely pathogenic variants (40 M/58 F). While detailed data are not available for every patient reported, 21 (18 M/3 F) developed hearing loss at as early as 3-months old (a male in family GR1.14) and five males had ocular lesions. Eleven patients had received a transplant (10 M/1 F), 22 patients (13 M/9 F) had impaired renal function and 15 patients (11 M/4 F) reached ESRD, with the earliest age being 17 years. Twelve variants are novel and presented for the first time in this work. Importantly, a high percentage of pathogenic variants (*n* = 5; 24%) are highly suspected to be de novo. It is expected that many more pathogenic variants will be found in the collagen IV genes as nearly every glycine is a candidate for a substitution that could lead to a recognisable phenotype.

With the inclusion of data from previous publications [18,42], we collectively studied the molecular genetics of 48 families with collagen IV nephropathies in the Greek population: 12 with defects in the autosomal *COL4A3/A4* genes diagnosed with TBMN and 36 AS families of the X-linked mode of inheritance. Regarding the X-linked families, in a total of 147 patients (55 M/92 F), 24 (18 M/6 F; 32.7%/6.5%) had reached ESRD. The relatively low percentage of males with ESRD reflects the young age of the included patients. Amongst female carriers, one reached ESRD at the young age of 23 years, which may be explained by skewed X-inactivation, as her mother and sister presented with a benign phenotype [18].

One of the pathogenic variants in the X-linked families, p.G624D, is a founder of global presence. Interestingly, we found this variant in nine non-related families from Greece who were geographically located in the northern, central and southern part of the country (Table 3). This is the most prevalent pathogenic variant amongst European populations, which was spread via migration during the 12th–13th centuries [21]. The frequency of this one variant alone in the gnomAD database is 1/5000 in non-Finnish Europeans, thereby implying that the disease is underdiagnosed and more prevalent than previously thought. Based on this, it is reasonable to hypothesize that many patients with atypical phenotypes are erroneously diagnosed and mistreated. Despite evidence that this variant is hypomorphic and associated with a much later age of onset of ESRD compared to patients with other *COL4A5* variants, there is clear variable expressivity that could be attributed to digenic inheritance or co-inheritance of random genetic modifiers [48,73]. In our cohort of 52 molecularly defined patients (21 M/31 F), 10 patients (6 M/4 F) developed CRF, two received a transplant and three had hearing loss. Only four males reached ESRD, at an average age of 49 years.

Finally, it is worth mentioning that although AS was previously known to be caused only by collagen IV DNA pathogenic variants, a recent publication may cast some doubt on this. Aypek et al. described three families with TBMN that carried recessive pathogenic variants in the *P3H2* (LEPREL1) gene, which encodes for prolyl 3-hydroxylase 2. This enzyme hydroxylates selected prolines of COL4 α chains at the 3′-position, in the context of Gly-3Hyp-4Hyp-Gly sequences post-translationally. The patients developed microscopic hematuria and microalbuminuria, as well as ocular symptoms including high-grade myopia, cataracts, and retinal detachment. At the ultrastructural level, one female patient showed TBMN and GBM irregularities reminiscent of AS. A conditional podocyte knockout mouse lacking the *P3h2* gene also exhibited AS-like features [19]. This discovery maintains AS as a collagen IV *protein* nephropathy [74].

## 5. Conclusions

NGS is very robust and enables molecular diagnosis when clinical or biochemical findings may be equivocal and lead to misdiagnosis. Familial segregation of microhematuria and proteinuria can result from defects in one of the *COL4A3/A4/A5* genes or in genes causing hereditary focal and segmental glomerulosclerosis or *MYH9*-related disorders. The effect that these defects have on medication, prognosis and family planning differ depending on depending on the affected gene. In the absence of overt family history, microhematuria may also be attributed to IgA nephropathy or else.

The early molecular confirmation of an AS diagnosis is of utmost importance, as it enables the use of RAAS blockade, thus avoiding potential erroneous and toxic immunosuppressive medication. This is especially true when the phenotype is atypical, such as in the case of pathogenic variant *COL4A5*-p.G624D, thus attaining precision nephrology. Furthermore, it empowers the participation of patients in clinical trials, as the correct diagnosis by molecular testing is imperative.

Molecular testing can resolve cases where the renal biopsy is unclear, especially in childhood where pathognomonic features of AS are not yet adequately established. Other examples of use include women presenting with microhematuria and low-grade proteinuria or young boys with *COL4A5* pathogenic variants who present with TBMN. A heterozygous variant in the *COL4A3/A4* is associated with a different risk of developing severe kidney disease compared to a hemizygous one in males or even a heterozygous change in the *COL4A5* gene in females.

The NGS approach inescapably leads to identification of multiple variants, the evaluation of which remains challenging, despite improvement of the tools used for their interpretation. To this end, the use of ACMG criteria, in silico algorithms and mining databases like HGMD and LOVD may be very beneficial.

## Figures and Tables

**Table 1:** Information on the 21 pathogenic variants identified and predictions of their potential effect in Greek families, using in silico analysis tools (like SIFT, PolyPhen-2, SNPs3D, Mutation Taster, Grantham score), searching in databases (like HGMD, LOVD, ARUP ALPORT, Ensembl, ClinVar), and taking in to account the classification according to ACMG criteria and their frequency (MAF). These pathogenic variants include: 10 missense, 2 nonsense, 2 frameshift, 3 exon-intron splicing and 4 gross deletions. Underlined are the 12 novel pathogenic variants detected in this study and not found in other families with familial hematuria or in healthy samples of the general population. These pathogenic variants were identified in 26 newly studied families from Greece, including one Cretan family. *: The following 3 families carrying the pathogenic variant COL4A5–p.G624D where previously published: GR1.27*and GR1.28* in Demosthenous P et al. 2012 and GR1.29 * in Pierides A et al. 2013. In family GR1.19, fourth from the end, an exon 37 deletion occurred. Inoue Y et al. 1999, reported a similar exon 37 deletion, with assumed different borderlines, classified as Pathogenic CG994851 [32]. ACMG classification criteria: PVS1-Pathogenic Very Strong, PS1-Pathogenic Strong 1, PM1-Pathogenic Moderate 1, PM2-Pathogenic Moderate 2, PM4-Pathogenic Moderate 4, PM5-Pathogenic Moderate 5, PP2-Pathogenic Supporting 2, PP3-Pathogenic Supporting 3, PP5-Pathogenic Strong 5. Abbreviations: GR, Greek origin; CR, Greek Cretan origin; del, deletion; MAF, Minor Allele Frequency; NA, Not Applicable.

Pathogenic Variant	FAMILY	ACMG Classification	HGMD	ClinVar	dpSNP	MAF	SIFT	PolyPhen-2	SNPs3D	Mutation Taster	Grantham Score	Align-GVGD	REVEL Score	References
*COL4A5* (ivs3)—c.232-2A > G	GR1.17	Pathogenic (PVS1, PM2, PP5)	NO DATA	ClinVar ID: 962843 Pathogenic	rs2065933012	NA	NA	NA	NA	Disease causing (Prob: 1)	NA	NA	NA—splice_acceptor_variant	[33]
*COL4A5* (ivs7)—c.438 + 5G > A	GR1.7	Likely Pathogenic (PM2, PM4)	Pathogenic CS107341	ClinVar ID: 24271 Pathogenic	rs281874739	NA	NA	NA	NA	Disease causing (Prob: 1)	NA	NA	NA - Splice_region, intron variant	[34,35,36]
*COL4A5*—c.973G > A, p.G325R	GR1.13	Pathogenic (PP5, PM1, PM2, PS1, PM5, PP2, PP3)	Pathogenic CM920213	ClinVar ID: 24352 Pathogenic	rs104886088	NA	Damaging (0)	Probably Damaging (1.000)	Deleterious (−3.04)	Disease causing (Prob: 0.999)	125	Class C65 (High)	Pathogenic (0.9879)	[11,37,38,39,40,41]
*COL4A5*—c.1129G > A, p.G377R	GR1.18	Pathogenic (PM1, PM2, PM5, PP2, PP3, PP5)	NO DATA	ClinVar ID: 818351 Likely Pathogenic	rs1603286154	NA	Damaging (0)	Probably Damaging (1.000)	Deleterious (−3.43)	Disease causing (Prob: 0.999)	125	Class C65 (High)	Pathogenic (0.9829)	This study
*COL4A5*—c.1402C > T, p.Q468 *	GR1.16	Pathogenic (PVS1, PM2, PP5)	NO DATA	ClinVar ID: 807569 Pathogenic	NA	NA	NA	NA	NA	Disease causing (Prob: 1)	NA	NA	NA— Stop gained	This study
*COL4A5*—c.1871G > A, p.G624D	GR1.21 GR1.22 GR1.23 GR1.24 GR1.25 GR1.26 GR1.27 * GR1.28 * GR1.29 *	Pathogenic (PP5, PM1, PM5, PP2, PP3)	Pathogenic CM983308	ClinVar ID: 24455 Pathogenic	rs104886142	A = 0.000087 (GnomAD_ exome)	Damaging (0.02)	Probably Damaging (0.999)	Deleterious (−2.62)	Disease causing automatic, (Prob: 0.999)	94	Class C65 (High)	Pathogenic (0.910)	[20,21,22,42,43,44,45,46,47,48,49,50,51,52]
*COL4A5*—c.2006G > A, p.G669D	GR1.4	Likely Pathogenic (PM1, PM2, PM5, PP2, PP3)	NO DATA	NA	NA	NA	Damaging (0.1)	Probably Damaging (1.000)	Deleterious (−3.45)	Disease causing (Prob: 0.999)	94	Class C65 (High)	Pathogenic (0.9779)	This study
*COL4A5*—c.2324G > A, p.G775D	GR1.20	Pathogenic (PM1, PM2, PP2, PP3)	Pathogenic CM115533	NA	NA	NA	Damaging (0)	Probably Damaging (1.000)	Deleterious (−3.60)	Disease causing (Prob: 0.999)	94	Class C65 (High)	Pathogenic (0.994)	[53]
*COL4A5* – c.2452_ 2454delATA/2653insT, p.I818Wfs*36	GR1.3	Likely Pathogenic (PVS1, PM2)	Pathogenic CX012578	ClinVar ID: 578169 Pathogenic	rs1569497013	NA	NA	NA	NA	Disease causing (Prob: 1)	NA	NA	NA— frameshift variant	[54]
*COL4A5* (ivs30)—c.2510-2A > G	GR1.2	Pathogenic (PVS1, PM2, PP5)	Pathogenic CS1934823	ClinVar ID: 562406 Pathogenic	rs760109866	G = 0.000006 (GnomAD_ exome)	NA	NA	NA	Disease causing (Prob: 1)	NA	NA	NA—splice_acceptor_variant	[46]
*COL4A5*—c.2719C > T, p.P907S	CR1.1	Likely Pathogenic (PM1, PM2, PP2)	NO DATA	NA	NA	NA	Tolerated (0.23)	Benign (0.193)	Deleterious (−0.39)	Disease causing (Prob: 0.960)	74	Class C65 (High)	Benign (0.456)	This study
*COL4A5*—c.2723G > A, p.G908E	GR1.6	Likely Pathogenic (PM1, PM2, PM5, PP2, PP3, PP5)	Pathogenic CM1822009	ClinVar ID: 235662 Likely pathogenic	rs878853089	NA	Damaging (0)	Probably Damaging (1.000)	Deleterious (−4.53)	Disease causing (Prob: 0.999)	98	Class C65 (High)	Pathogenic (0.990)	[55,56]
*COL4A5*—c.3044G > A, p.G1015E	GR1.8	Likely Pathogenic (PM2, PM1, PM5, PP2, PP3, PP5)	Pathogenic CM960379	NA	NA	NA	Damaging (0)	Probably Damaging (1.000)	Deleterious (−4.27)	Disease causing (Prob: 0.999)	98	Class C65 (High)	Pathogenic (0.9869)	[57,58]
*COL4A5*—c.3772G > A, p.G1258S	GR1.5	Likely Pathogenic (PM2, PM1, PP2, PP3, PP5)	NO DATA	ClinVar ID: 807396 Pathogenic	NA	NA	Damaging (0.01)	Probably Damaging (1.000)	Deleterious (−3.30)	Disease causing (Prob: 0.999)	56	Class C55	Pathogenic (0.9739)	This study
*COL4A5*—c.4099C > T, p.Q1367*	GR1.15	Likely Pathogenic (PVS1, PM2)	NO DATA	NA	NA	NA	NA	NA	NA	Disease causing (Prob: 1)	NA	NA	NA— Stop gained	This study
*COL4A5*—c.4382_4383 insCATGG, p.F1462Mfs*88	GR1.12	Pathogenic (PVS1, PM2)	NO DATA	NA	NA	NA	NA	NA	NA	Disease causing (Prob: 1)	NA	NA	NA— frameshift variant	This study
*COL4A5*—c.4428C > G, p.C1476W	GR1.11	Likely Pathogenic (PM5, PM1, PM2, PP2, PP3, PP5)	NO DATA	NA	NA	NA	Damaging (0)	Probably Damaging (1.000)	Deleterious (−3.17)	Disease causing (Prob: 0.999)	215	Class C65 (High)	Pathogenic (0.8299)	This study
*COL4A5* del exon 37	GR1.19	NA	NO DATA	NA	NA	NA	NA	NA	NA	NA	NA	NA	NA	This study
*COL4A5* del whole exon41	GR1.14	NA	NO DATA	NA	NA	NA	NA	NA	NA	NA	NA	NA	NA	This study
*COL4A5* del ivs1-ivs29	GR1.9	NA	NO DATA	NA	NA	NA	NA	NA	NA	NA	NA	NA	NA	This study
*COL4A5* del ivs1-ivs41	GR1.10	NA	NO DATA	NA	NA	NA	NA	NA	NA	NA	NA	NA	NA	This study

**Table 2:** Demographic, clinical, pathologic and mutational analysis data for the 26 families described herein. All families had been referred for a diagnosis because of the presence of familial microscopic hematuria and/or suspicion of Alport syndrome. Some families had many more members than DNA available. Abbreviations: MH, Microscopic hematuria; GFR, Glomerular filtration rate; CRF, Chronic Renal Failure; ESRD, End-stage renal disease; Bx, Biopsy; LM, Light Microscope; EM, Electron Microscope; FSGS, Focal segmental glomerulosclerosis; GBM, Glomerular Basement Membrane; TBMN, Thin basement membrane nephropathy; AS, Alport Syndrome; Tx, Transplant; yo, years old; GR, Greek origin; CR, Greek Cretan origin.

Family	Pathogenic Variant Carriers (Molecularly Confirmed)			Biopsy Results & Comments	Pathogenic Variant	Hearing Loss	Ocular Lesions	Tx (Age)	MH Only (Age)	MH + Proteinuria, Normal GFR (Age)	Impaired Renal Function, CRF or ESRD (Age)	ESRD (Age)
	Total	♂	♀									
**CR1.1**	4	0	4	1♀ (20-yo): GBM defects pathognomonic for AS EM: thickened with irregular textured lamina densa in glomeruli, lamellation, focally fused podocyte foot processes 2♂ historical (uncles II.1 & II.2) biopsies, deceased with AS. The 2♂ reached ESRD at 15-yo & 19-yo, both received a Tx. Their grandmother (I.2) also received a Tx at 58-yo.	*COL4A5*- c.2719C > T, p.P907S	-	-	1♀ (49-yo)	3♀ (infancy)	-	1♀ (39-yo)	1♀ (39-yo)
**GR1.2**	3	1	2	No biopsy The 1♂ received a Tx at 28-yo, has 1 brother (no DNA available, II.3) with ESRD at 20-yo	*COL4A5* (ivs30)- c.2510-2A > G	-	-	1♂ (28-yo)	-	1♀ (7-yo)	1♂ (26-yo) 1♀	1♂ (26-yo)
**GR1.21**	9	4	5	1♂ (19-yo) only LM—no pathological lesions other than thin GBM	*COL4A5*- c.1871G > A, p.G624D	-	-	1♂	2♂, 4♀	1♂ (39-yo)	1♂ (51-yo)	1♂ (51-yo)
**GR1.22**	2	0	2	No biopsy 1♀ (38-yo & 45-yo), nephrograms: small kidneys 1♂ historical (father I.1) biopsy (no DNA available) diagnosed chronic glomerulonephritis.	*COL4A5*- c.1871G > A p.G624D	-	-	-	1♀ (14yo)	-	1♀ (45-yo)	-
**GR1.23**	4	1	3	1♀ (43-yo), a few lesions of mesangial proliferative glomerulonephritis	*COL4A5*- c.1871G > A, p.G624D	-	-	-	1♂, 1♀	1♀	-	-
**GR1.3**	9	4	5	No biopsy 3 ♂ members reached ESRD and revied a Tx, presenting also hearing problems	*COL4A5*- c.2452-2454delATA/2653insT, p.I818Wfs*36	4♂	3♂	3♂ (36-yo rejected 16-y later, 26-yo, 25-yo)	-	1♂ 4♀ (3 at 31-yo, 33-yo, 35-yo)	3♂ (24-yo, 25-yo, ~36-yo), 1♀ (41-yo)	3♂ (24-yo, 25-yo, ~36-yo) 1♀ (41-yo)
**GR1.4**	6	2	4	No biopsy	*COL4A5*- c.2006G > A, p.G669D	1♂ (8-yo)	-	-	3♀	2♂	-	-
**GR1.5**	6	3	3	Biopsies of 3♂ members Proband 1♂ (III.1), Bx-No EM: FSGS/Nephrotic Syndrome Half-brother 1♂ (III.3), Bx (20-yo): FSGS, EM: thickening & rarely thinning of GBM, podocyte foot process effacement Half-brother 1♂ (III.4), Bx (16-yo): FSGS,	*COL4A5*- c.3772G > A, p.G1258S	2♂, 1♀	-	-	2♀	2♂	1♂, 1♀	1♂
**GR1.6**	2	1	1	No biopsy The uncle of proband (no DNA available, II.3) with sensorineural hearing loss, received a Tx at 18-yo.	*COL4A5*- c.2723G > A, p.G908E	1♂ (7-yo)	-	-	1♀	1♂ (7-yo)	-	-
**GR1.7**	5	3	2	1♀ (30-yo), Non-specific findings, minimum mesangial proliferative alterations, negative in deposition of immune complexes. Her 46-yo brother (III.1), uncle of the proband, after reaching ESRD, received a graft with membranous nephropathy.	*COL4A5* (ivs7)- c.438 + 5G > A	-	-	1♂	2♀	1♂ (3.5-yo)	1♂	1♂
**GR1.24**	10	7	3	1♂, (35-yo): in ≥38% focal ischemic lesions of glomeruli & atrophic tubules. Most glomeruli display mild focal thickening of Bowman’s capsule (BC), while others show a moderate degree of ischemia with fibrous thickening of the BC and some display total sclerosis with intracapsular fibrosis. A subject with sensorineural hearing loss presents gradual deterioration of kidney function that stabilizes when treated with statins & ACE-i.	*COL4A5*- c.1871G > A, p.G624D	1♂	-	-	3 ♂, 3♀	2♂	1♂	-
**GR1.8**	2	1	1	No biopsy	*COL4A5*- c.3044G > A, p.G1015E	1♂, 1♀	1♂	-	1♀	1♂ (24-yo)	-	-
**GR1.9**	4	3	1	1♂, Bx (26-yo): crescent formations in about 40% and IgG & C3 deposits Initial erroneous diagnosis of rapidly progressive glomerulonephritis Nonresponsive to immunosuppressive treatment with corticosteroids and cyclophosphamide, started HD 1 year later (27-yo). He received a Tx at 27-yo, receiving a graft from his sister (II.2, No MH) but rejected it 3 years later, resulting in HD (at 30-yo) until his death, 52-yo. Diagnosis changed 4 years later (30-yo), suspecting a hereditary renal disease, AS, after identifying his 17-yo and 19-yo nephews. with CRF and then ESRD. These were the 2 sons of his sister (III.2, III.3), that received a Tx at 21-yo (2nd Tx at 28-yo) and 32-yo respectively. All 3♂ had sensorineural hearing loss.	*COL4A5*- del ivs1-ivs29	3♂	-	3♂ (1♂Tx: 21-yo, 1♂ 1st Tx: 21-yo, 2nd Tx: 28yo, 1♂Tx: 32-yo)	-	-	3♂, (17-yo, 19-yo, 27-yo)	3♂, (17-yo, 19-yo, 27-yo)
**GR1.10**	2	0	2	1♀, 1st Bx (5-yo): mesangial proliferative glomerulonephritis 2nd Bx (11-yo): suspicion for IgA nephropathy	*COL4A5*- del ivs1-ivs41 probably *de novo*	-	-	-	1♀ (infancy)	1♀	-	-
**GR1.11**	5	1	4	No biopsy	*COL4A5*- c.4428C > G, p.C1476W	-	-	1♂ (23-yo) Preparing for 2nd Tx	1♀ (5mo)	1♀ (14-yo)	1♂ (23-yo)	1♂ (23-yo)
**GR1.12**	1	0	1	1♀, normal immunological staining, normal ultrasound of the kidney & normal Doppler of renal vessels	*COL4A5*- c.4382_4383 insCATGG p.F1462Mfs*88 *de novo*	-	-	-	-	1♀ (8,5-yo)	-	-
**GR1.25**	4	1	3	1♀ (41-yo): suspicion for AS/TBMN, negative immunostaining, EM: thinning & lamination in parts of GBM (TBMN) & podocyte foot process effacement	*COL4A5*- c.1871G > A, p.G624D	-	-	-	1♀ (14-yo)	-	2♀ (1♀, 39-yo)	-
**GR1.13**	3	1	2	1♂, 2nd Bx (38-yo): glomerulopathy & elements with secondary FSGS	*COL4A5*- c.973G > A, p.G325R	-	-	-	1♀ (1.5-yo)		1♂ (34-yo), 1♀	1♀
**GR1.14**	2	1	1	No biopsy	*COL4A5*- del exon 41 probably *de novo*	1♂ (3-mo)	-	-	1♀	1♂ (9-yo)	-	-
**GR1.15**	2	1	1	1♂, No EM, no pathognomonic findings	*COL4A5*- c.4099C > T, p.Q1367*	1♂ (11yo)	-	-	-	1♂ (9-yo)	-	-
**GR1.16**	4	1	3	1♀, Bx (43-yo): Non-immune complex glomerulonephritis LM: FSGS with partial tubular atrophy (15%) EM: slight thickening of some GBM, segmental effacement of podocyte foot processes	*COL4A5*- c.1402C > T, p.Q468*	1♂, 1♀	1♂	-	-	1♂, 2♀ (1♀, 4-yo)	1♀ (54-yo)	1♀ (54-yo)
**GR1.26**	1	1	0	No biopsy	*COL4A5*- c.1871G > A, p.G624D	-	-	-	-	1♂	-	-
**GR1.17**	4	1	3	No biopsy	*COL4A5* (ivs3)- c.232-2A > G	1♂	-	-	2♀ (10-mo, 10-yo)	1♂ (34-yo), 1♀	-	-
**GR1.18**	1	1	0	1♂ Bx (13-yo): Glomerular membrane defects pathognomonic of AS EM: irregular alternation & thinning of GBM segments, mild & focal mesangial hyperplastic lesions, segmental effacement of podocyte foot processes	*COL4A5*- c.1129G > A, p. G377R *de novo*	1♂ (14-yo)	-	-	-	1♂ (12-yo)	-	-
**GR1.19**	2	1	1	No biopsy	*COL4A5*- del exon 37	-	-	-	-	1♂ (22-mo)	-	-
**GR1.20**	1	0	1	1♀ Bx (10-yo): suspicion of TBMN or AS EM: mainly thinning of GBM, mild & focal mesangial hyperplastic lesions, segmental effacement of podocyte foot processes, Negative immunological test	*COL4A5*- c.2324G > A, p.G775D *de novo*	-	-	-	-	1♀ (9-yo)	-	-
**SUM (%)**	**98** **100%**	**40**♂ **41%**	**58**♀ **59%**	**17 (17,3%) in 15 families** **(9**♂**/8**♀**)**	**21 different pathogenic variants**	**21** (**18**♂**/3**♀**)**	**5** **5**♂	**11** **(10**♂**/1**♀**)** **11.2%**	**34** **(6**♂**/28** ♀**)** **34.7%**	**31** **(18**♂**/13**♀**)** **31.6%**	**22** **(13**♂**/9**♀**)** **22.4%**	**15** **(11**♂**/4**♀**)** **15.3%**

**Table 3:** Data for nine families with the founder pathogenic variant *COL4A5*-c.1871G > A, p.G624D. Fifty-two members tested positive (21 males and 31 females). The described patients belong to the six families mentioned here (GR1.21, GR1.22, GR1.23, GR1.24, GR1.25, GR1.26) and another three families, which were mentioned in previous publications (GR1.27 * & GR1.28 * in Demosthenous P et al. 2012 and GR1.29 * in Pierides A et al. 2013). Abbreviations: MH, Microscopic hematuria; GFR, Glomerular filtration rate; CRF, Chronic Renal Failure; ESRD, End-stage renal disease; Bx, Biopsy; LM, Light Microscope; EM, Electron Microscope; FSGS, Focal segmental glomerulosclerosis; GBM, Glomerular basement membrane; TBMN, Thin basement membrane nephropathy; AS, Alport Syndrome; Tx, Transplanted; yo, years old; GR, Greek origin.

Family	Pathogenic Variant Carriers (Molecularly Confirmed)			Biopsy Results/Comments	HEARING LOSS	Ocular Lesions	Tx (Age)	MH Only (Age)	MH+ Proteinuria, Normal GFR (Age)	Impaired Renal Function, CRF or ESRD (Age)	ESRD (Age)
	Total	♂	♀								
**GR1.21** (Athens)	9	4	5	1♂ (19-yo) only LM—no pathological lesions other than thin GBM, possible TBMN or GBM glomerulonephritis	-	-	1♂	2♂, 4♀	1♂ (39-yo)	1♂ (51-yo)	1♂ (51-yo)
**GR1.22** (Thessaloniki)	2	0	2	No biopsy 1♀ (38-yo & 45-yo), nephrograms: small kidneys 1♂ historical (father I.1) biopsy (no DNA available, I.1) diagnosed chronic glomerulonephritis.	-	-	-	1♀ (14-yo)	-	1♀ (45-yo)	-
**GR1.23** (Patra)	4	1	3	1♀ (43-yo), a few lesions of mesangioproliferative glomerulonephritis	-	-	-	1♂, 1♀	1♀	-	-
**GR1.24** (Ioannina)	10	7	3	1♂, (35-yo): in ≥38% focal ischemic lesions of glomeruli & atrophic alterations of tubules. Most of the glomeruli display mild focal thickening of Bowman’s capsule (BC), while others display a moderate degree of ischemia with fibrous thickening of the BC and some display total sclerosis with intracapsular fibrosis. The subject with sensorineural hearing loss presents gradual deterioration of kidney function that stabilizes when treated with statins & ACE-i.	1♂	-	-	3♂, 3♀	2♂	1♂	-
**GR1.25** (Ioannina)	4	1	3	1♀ (41-yo): suspicion for AS/TBMN, Negative immunostaining, EM: thinning & lamination in parts of GBM (TBMN) & podocyte foot process effacement	-	-	-	1♀ (14-yo)	-	2♀ (1♀, 39-yo)	-
**GR1.26** (Volos)	1	1	0	No biopsy	-	-	-	-	1♂	-	-
GR1.27 * (Thessaloniki) Demosthenous P et al. 2012	7	3	4	No biopsy	2♂	-	-	-	2♂, 4♀	1♂ (39-yo)	1♂ (39-yo)
GR1.28 * (Athens) Demosthenous P et al. 2012	5	2	3	1♂, (51-yo): diagnosed FSGS 1♂, (47-yo): diagnosed AS, LM: diffused glomerulosclerosis with chronic interstitial changes, tubular atrophy and fibrosis EM: FSGS & thin GBM in all areas except where it splits focally into many layers, also splitting of BM in tubules	-	-	1♂ (52-yo)	1♀ (20s-yo)	1♀ (30-yo)	2♂ (1♂, 46-yo) 1♀	1♂ (46-yo)
GR1.29 * (Patra) Pierides A et al. 2013	10	2	8	No biopsy	-	-	-	1♂, 5♀	1♀	1♂ (61-yo)	1♂ (61-yo)
**TOTAL**	**52**	**21**♂	**31**♀	**6 in 5 families** **4**♂**/2**♀	**3** **3**♂	**-**	**2** **2**♂	**23** **7**♂**/16**♀ **44.2%**	**13** **6**♂**/7**♀ **25%**	**10** **6**♂**/4**♀ **19.2%**	**4** **4**♂ **7.7%**

## Supplementary Materials

The following supporting information can be downloaded at: https://www.mdpi.com/article/10.3390/genes13122203/s1, Figure S1: (S1.1–S1.29). Pedigrees of the 29 families with a pathogenic variant in the X-linked *COL4A5* gene described in this study. The pedigrees of the nine families (six studied here and three mentioned in previous publications, indicated with an asterisk*) with the founder pathogenic variant *COL4A5*-c.1871G > A, p.G624D, are depicted at the end. We included mostly subjects with DNA available for the diagnosis plus some healthy subjects to provide a better overall pattern of inheritance. Patients filled in light green have been confirmed molecularly. Patients with symptoms but with no available DNA and/or who are obligate carriers are represented with grey filled symbols in the pedigrees. Each subject is represented by a pedigree ID number, which includes a roman numeral for the generation and an Arabic number, a genotype if it is molecularly confirmed and some distinct clinical data. To manage family and clinical data to create the above pedigrees we used PhenoTips (REF https://pubmed.ncbi.nlm.nih.gov/23636887/ accessed on 13 October 2022) that uses “Open-Pedigree”, an open-source tool, as its pedigree editor. Abbreviations: d.; died; yo, years old; mo, months; wk, weeks; E +, positive evaluation through genetic testing; E-, negative evaluation through genetic testing; HET, heterozygous; HEM, hemizygous; NEG, negative; MH, Microscopic hematuria; GFR, Glomerular filtration rate; CRF, Chronic renal failure; ESRD, End-stage renal disease; HD, hemodialysis; Bx, Biopsy; LM, Light Microscope; EM, Electron Microscope; FSGS, Focal segmental glomerulosclerosis; GBM, Glomerular basement membrane; TBMN, Thin basement membrane nephropathy; HP, hypertension; AS, Alport Syndrome; Tx, Transplanted; GR, Greek origin; CR, Greek Cretan origin; ?, no data. Table S1: Information on the experimental conditions and reagents used for the verification by PCR and Sanger DNA sequencing of the 17 of 21 mutations discovered through NGS in this study. Underlined are 8 novel point mutations identified here. If no restriction enzyme was available for the examination of additional samples, the identification of the mutation was performed by direct Sanger DNA sequencing. Four other structural mutations, involving deletions, reported in this work were identified with the use of MLPA analysis.
