# Supplementary material for: Novel and Founder Pathogenic Variants in X-Linked Alport Syndrome Families in Greece
Source: Genes (Basel). 2022 Nov 24;13(12):2203. doi: 10.3390/genes13122203 (PMC9778032; doi:10.3390/genes13122203)
Supplement: Supplementary file 1 [file genes-13-02203-s001.zip › Figure S1.pdf]

S1.1 Family CR1.1

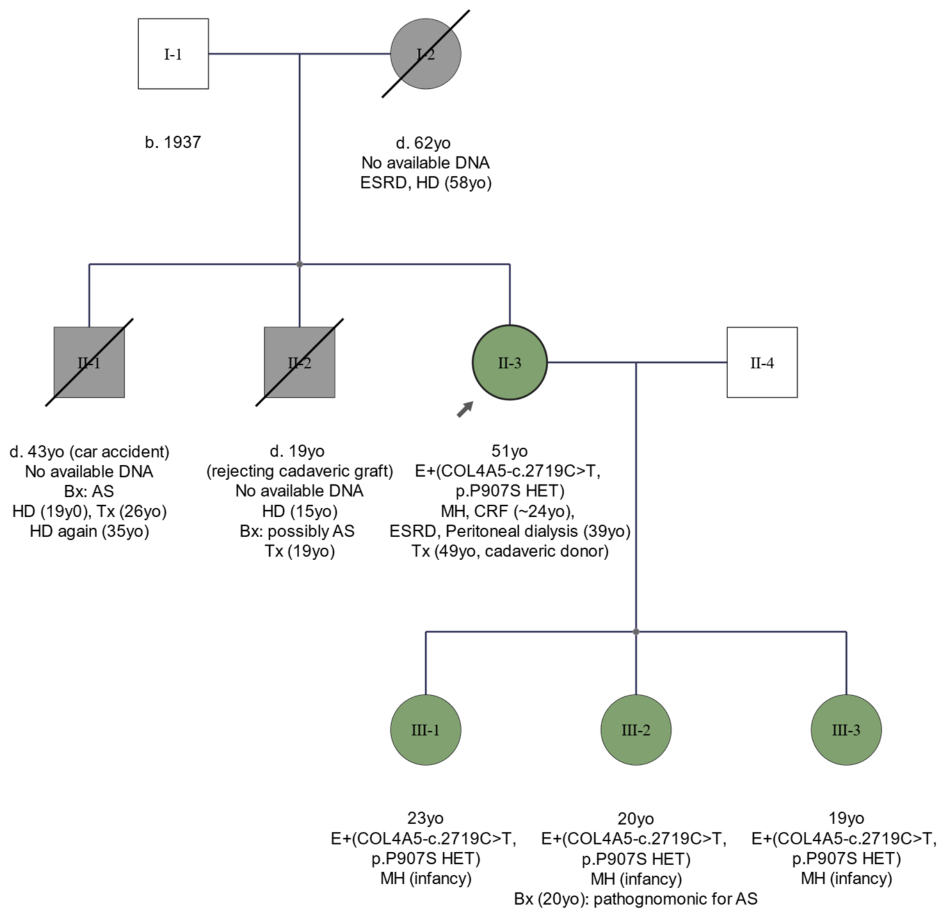

S1.2 Family GR1.2

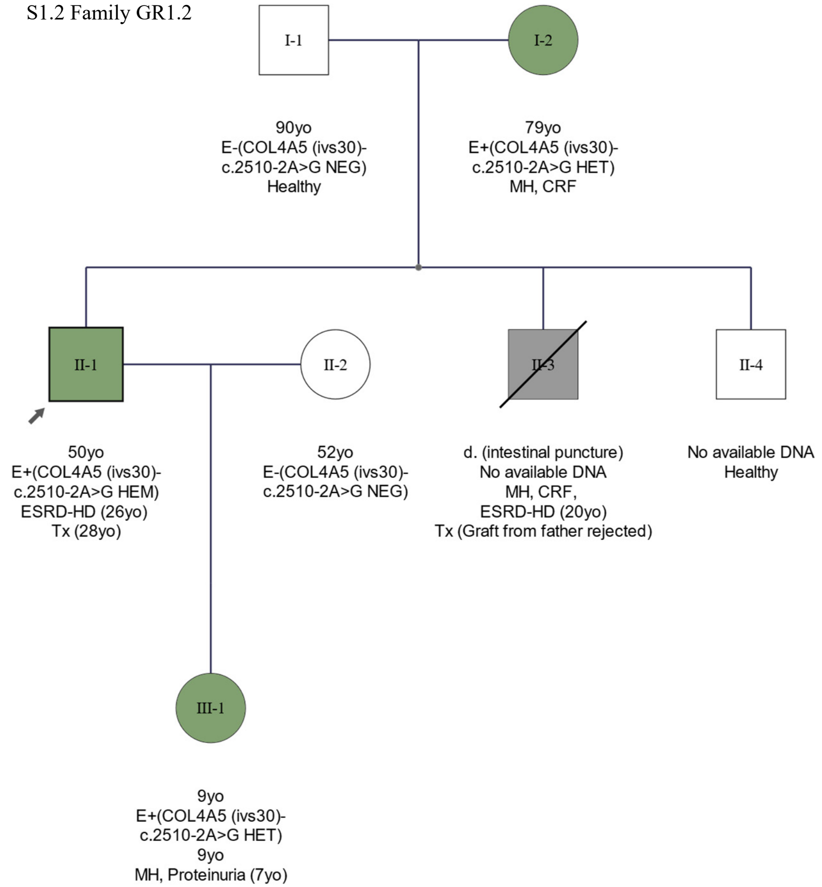

S1.3 Family GR1.3

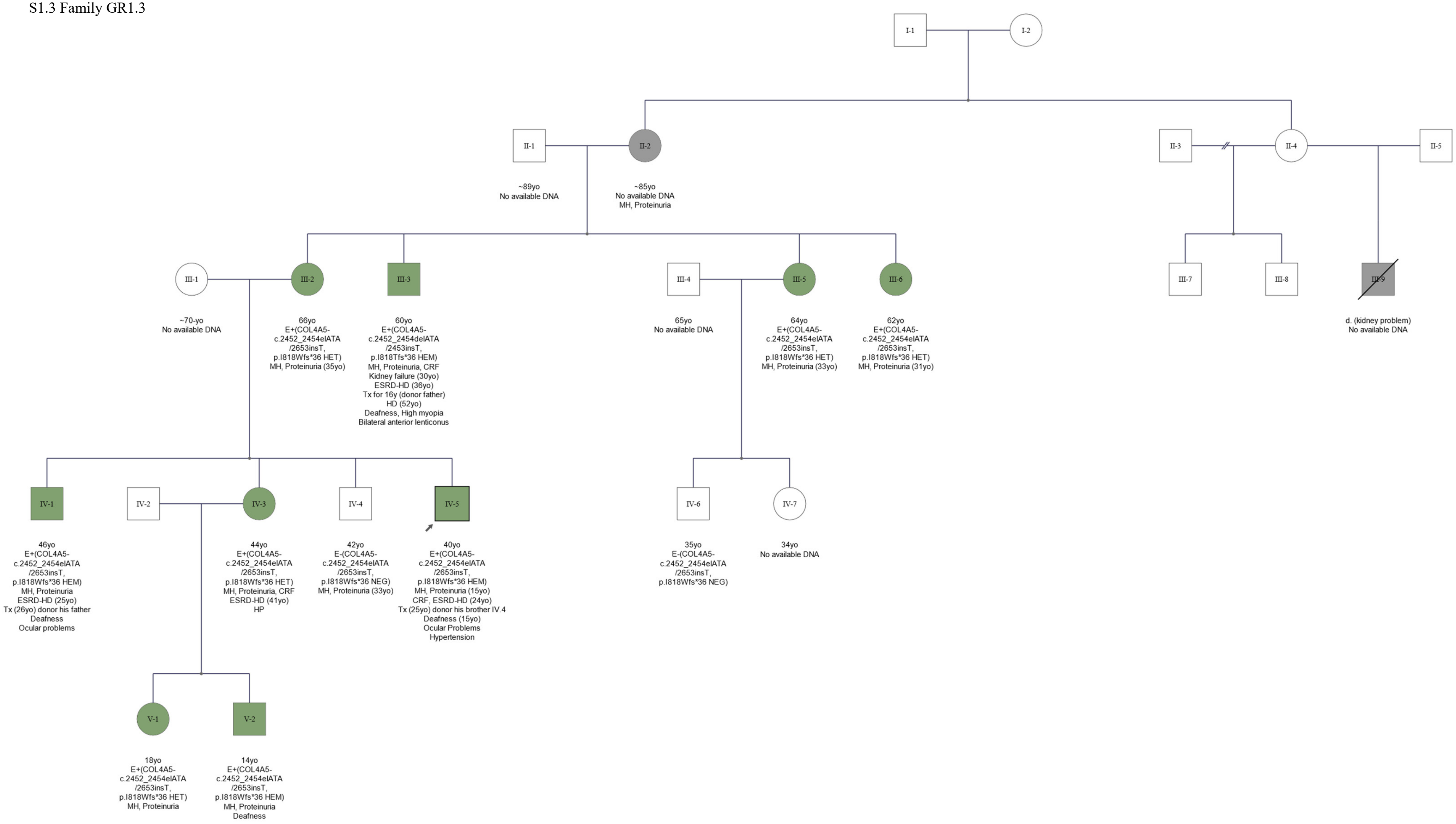

S1.4 Family GR1.4

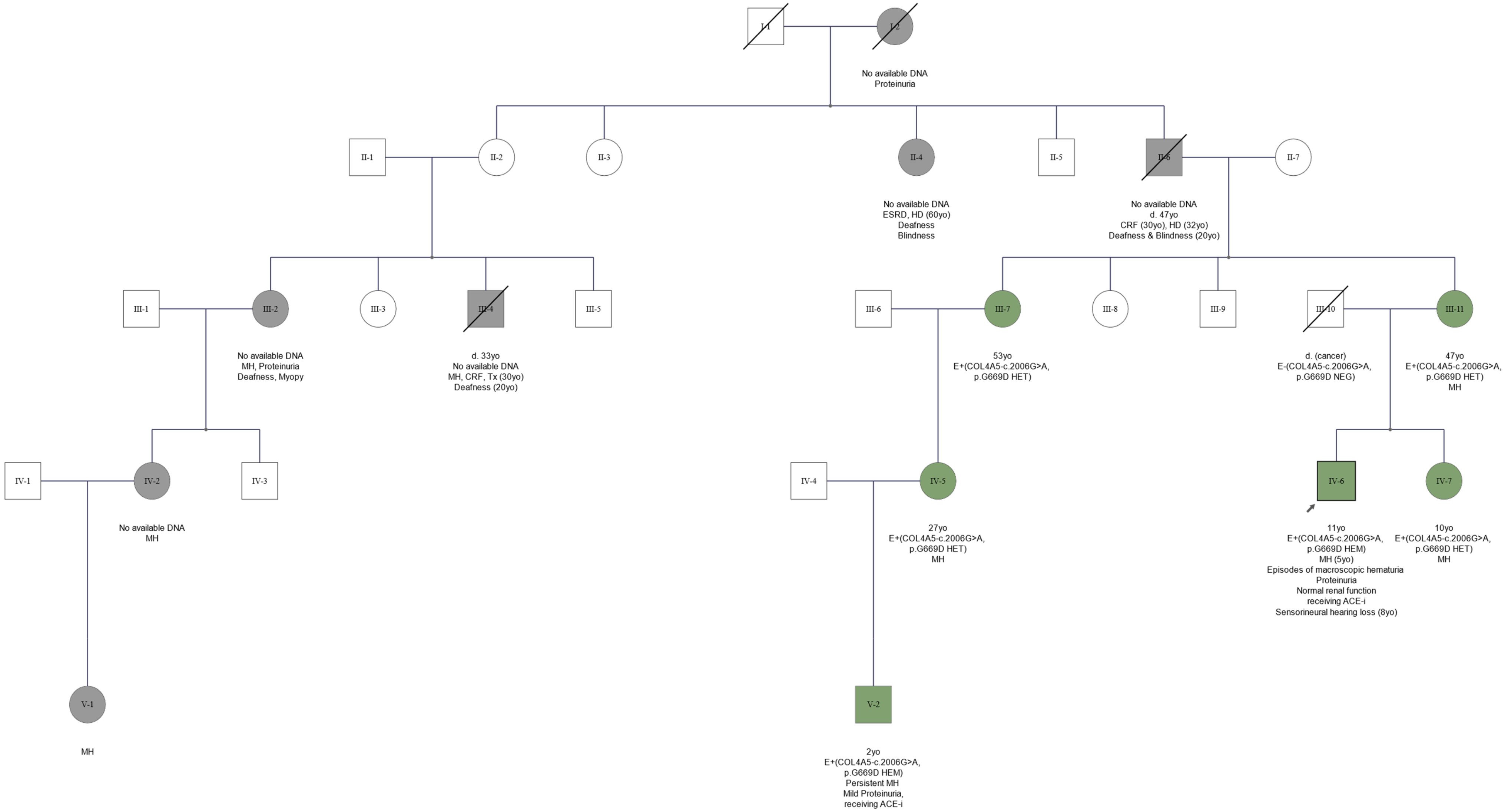

S1.5 Family GR1.5

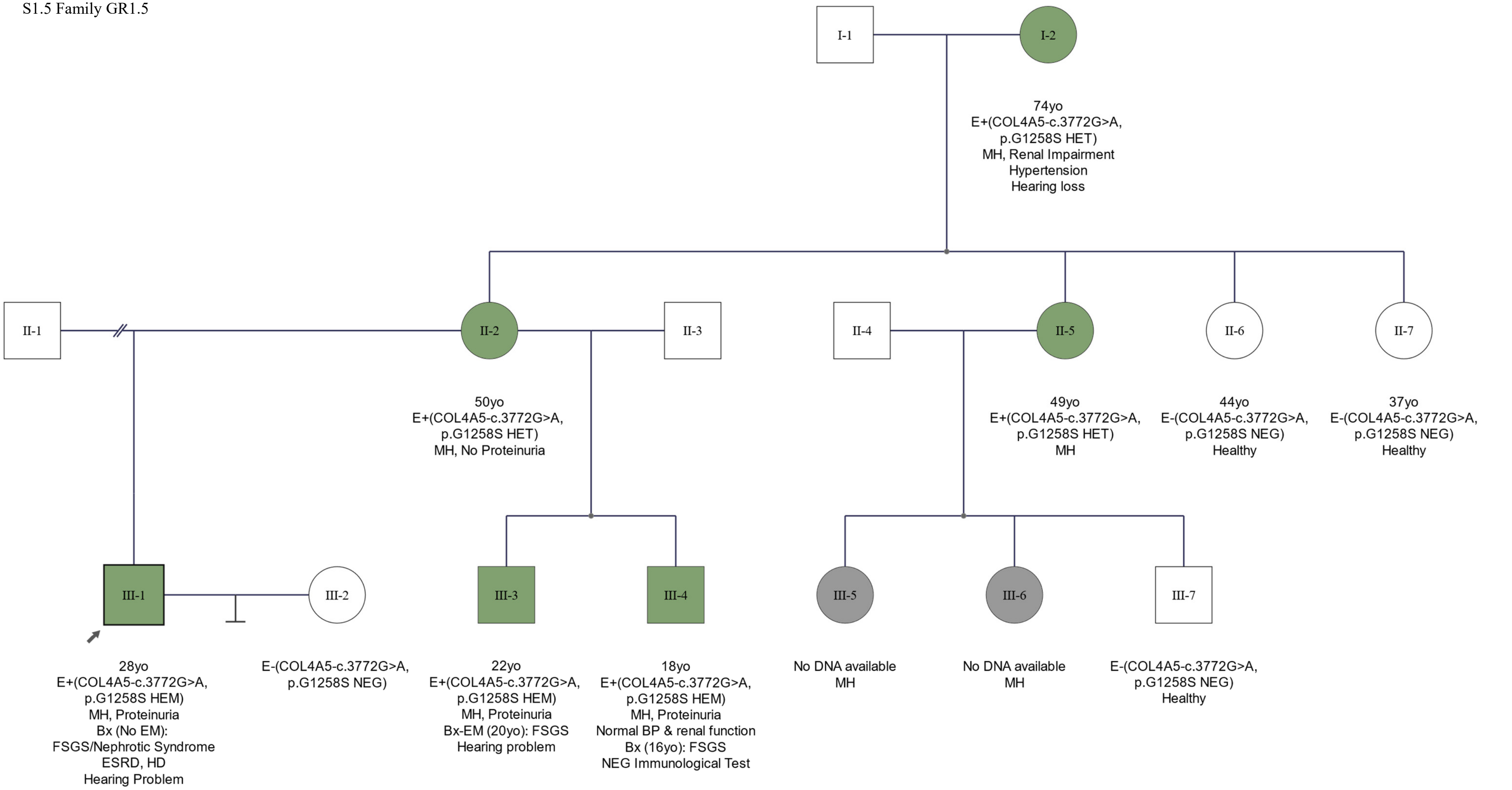

S1.6 Family GR1.6

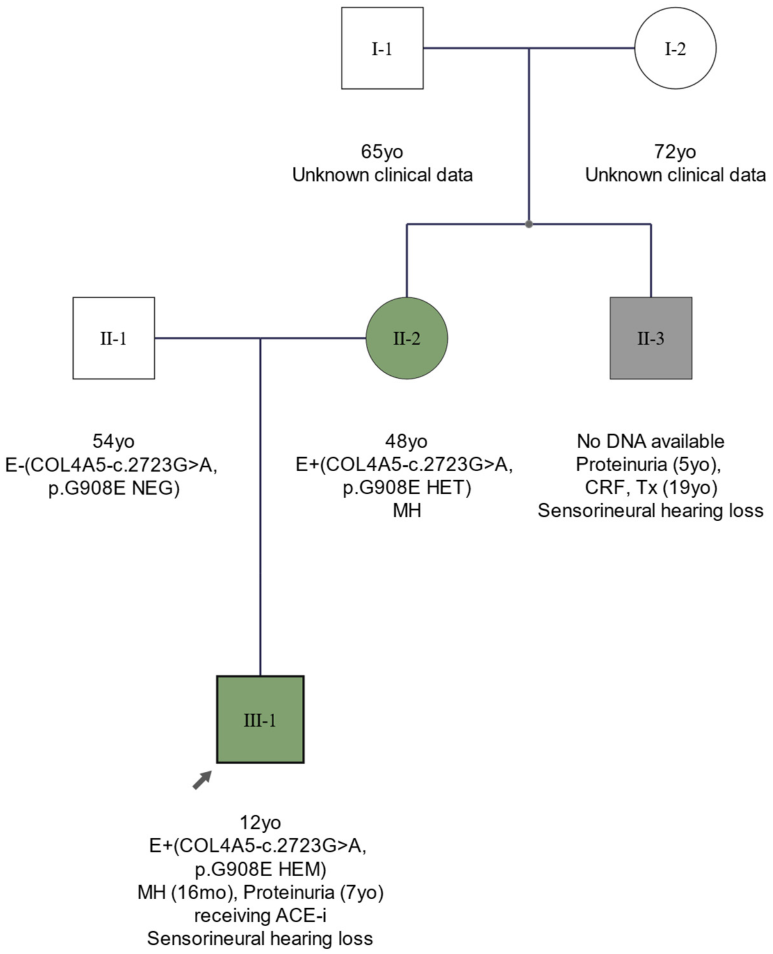

S1.7 Family GR1.7

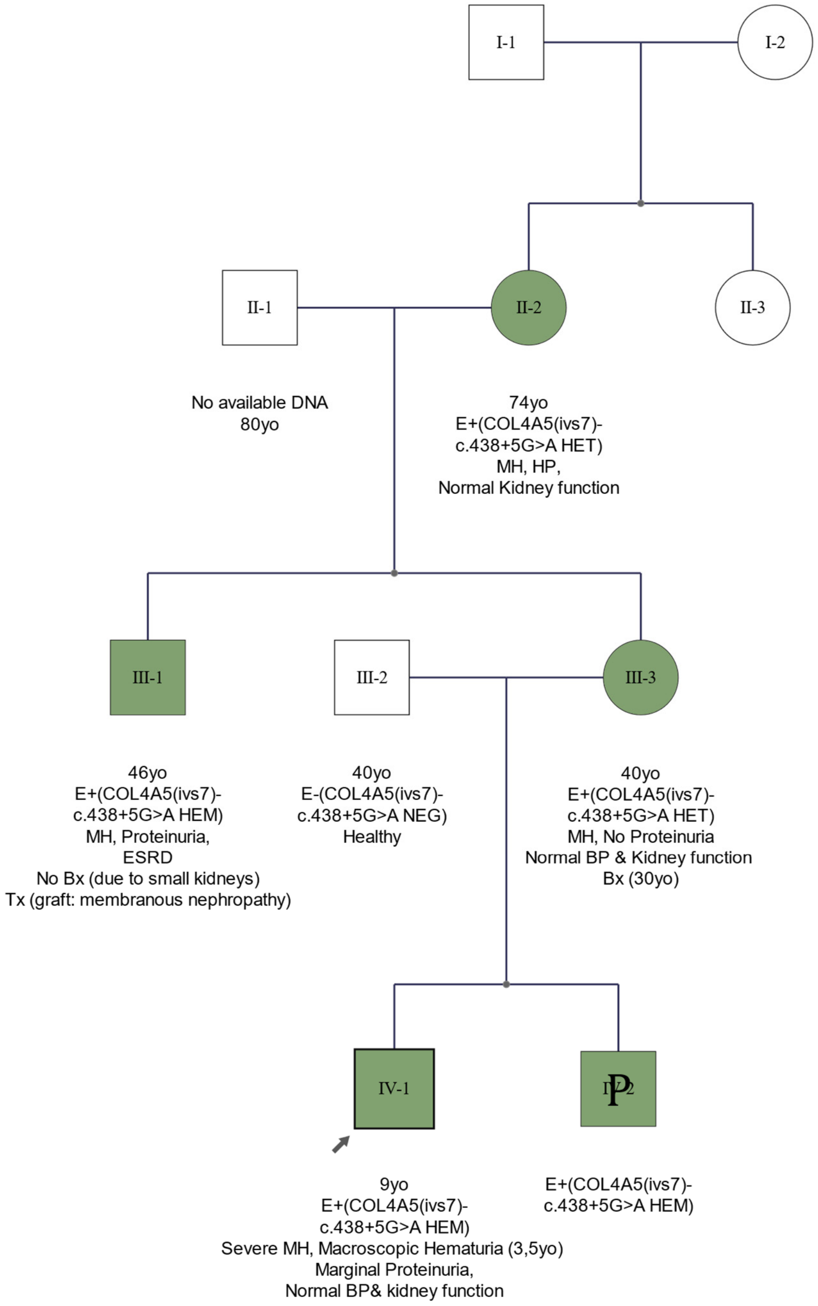

S1.8 Family GR1.8

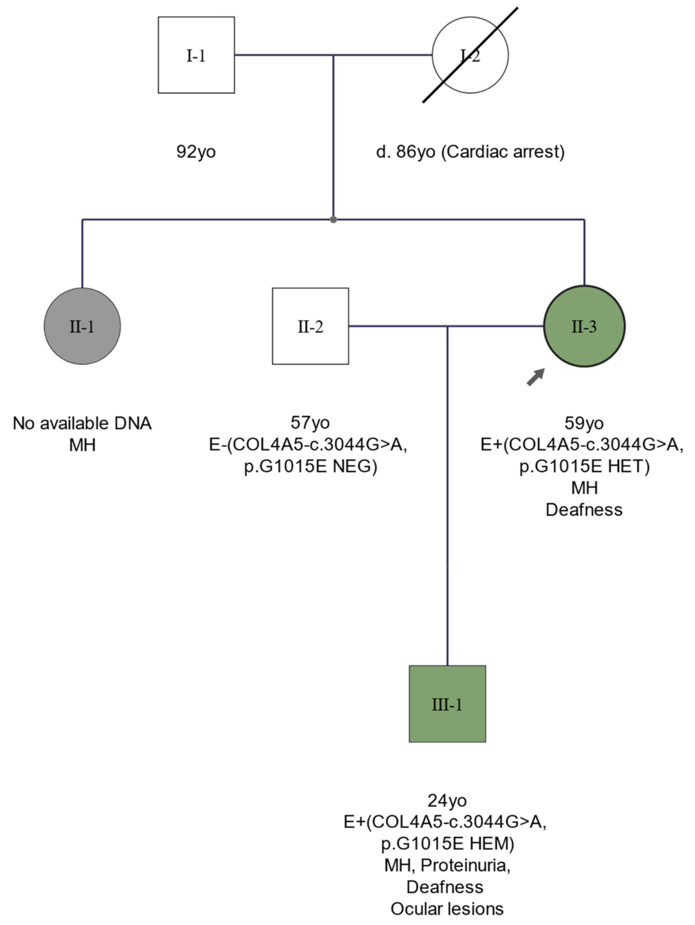

S1.9 Family GR1.9

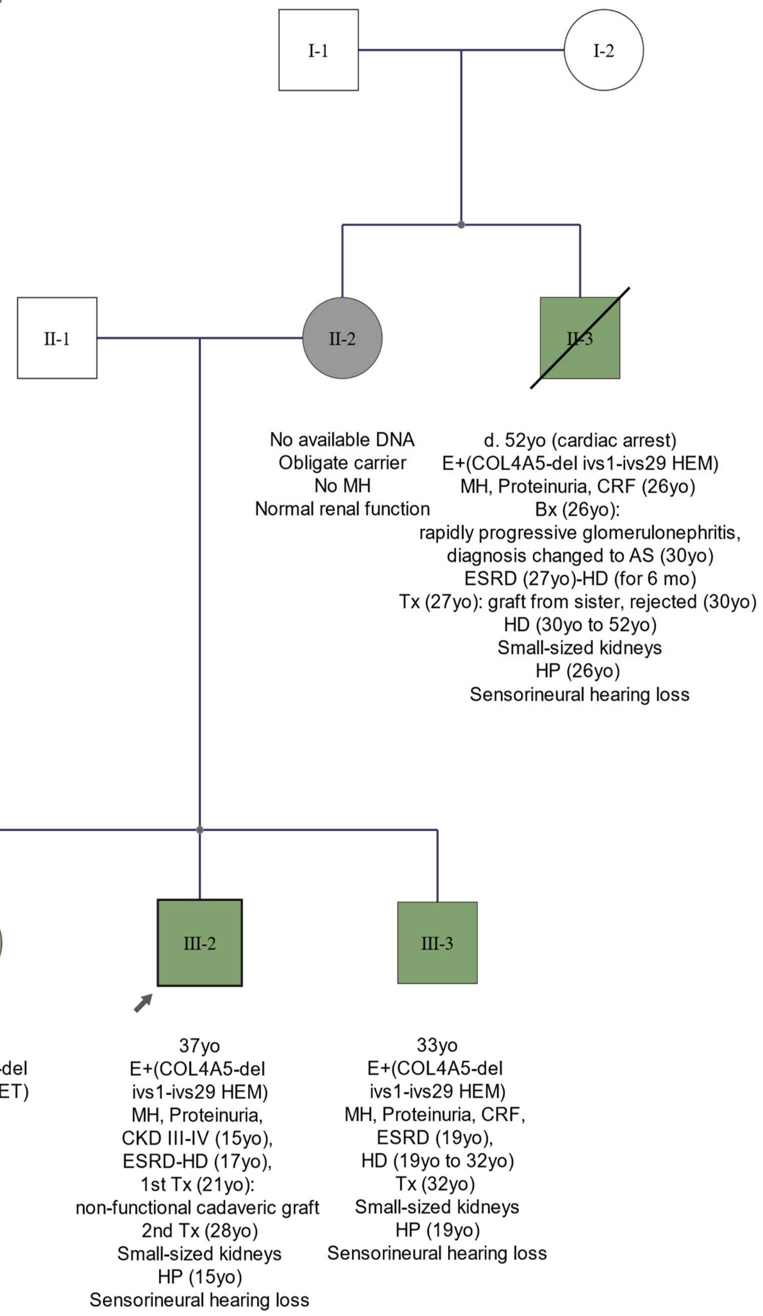

S1.10 Family GR1.10

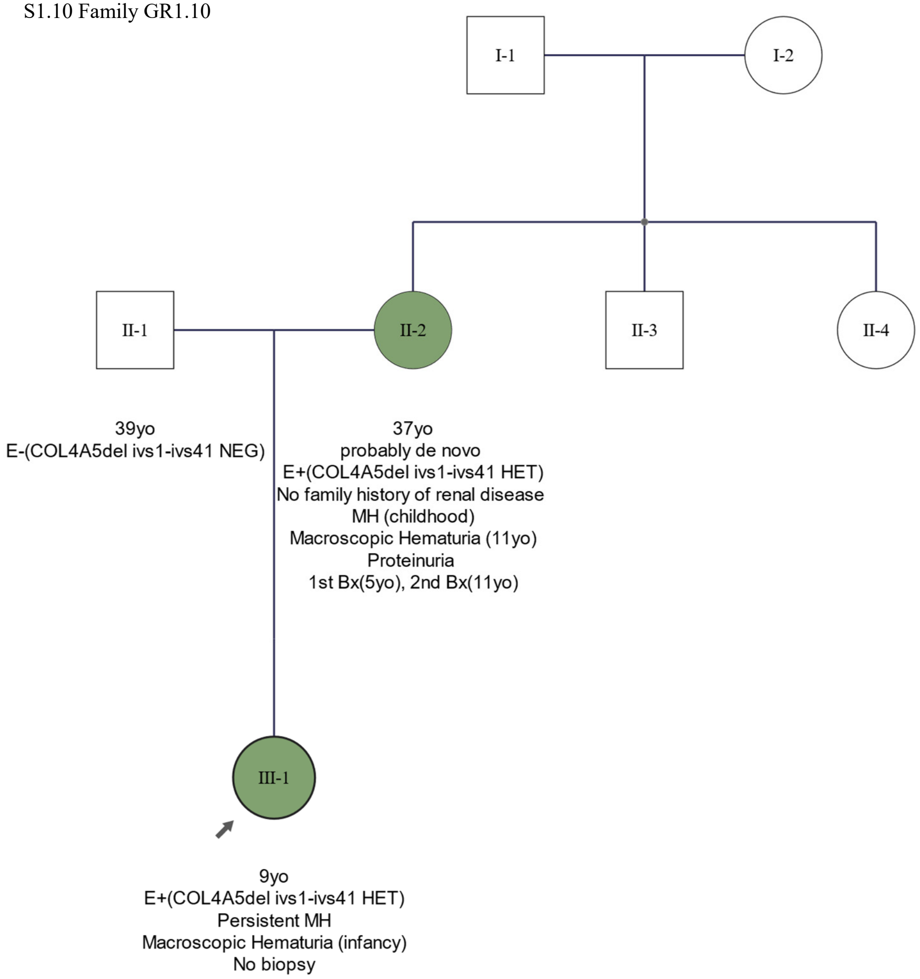

S1.11 Family GR1.11

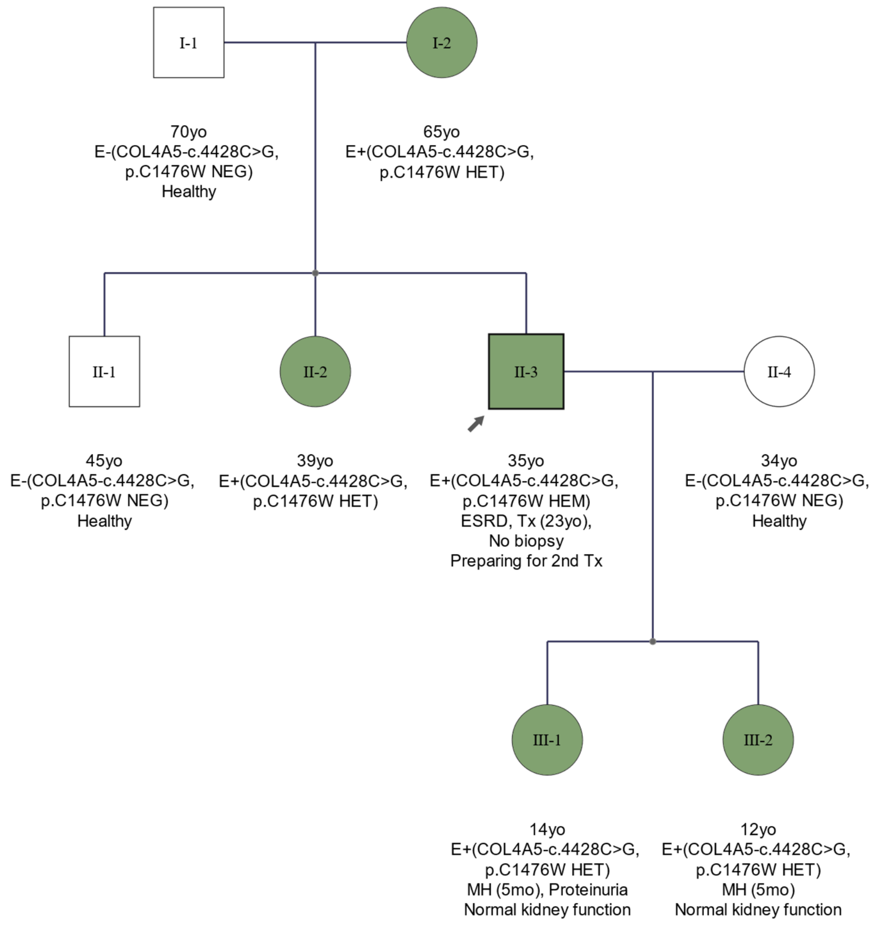

S1.12 Family GR1.12

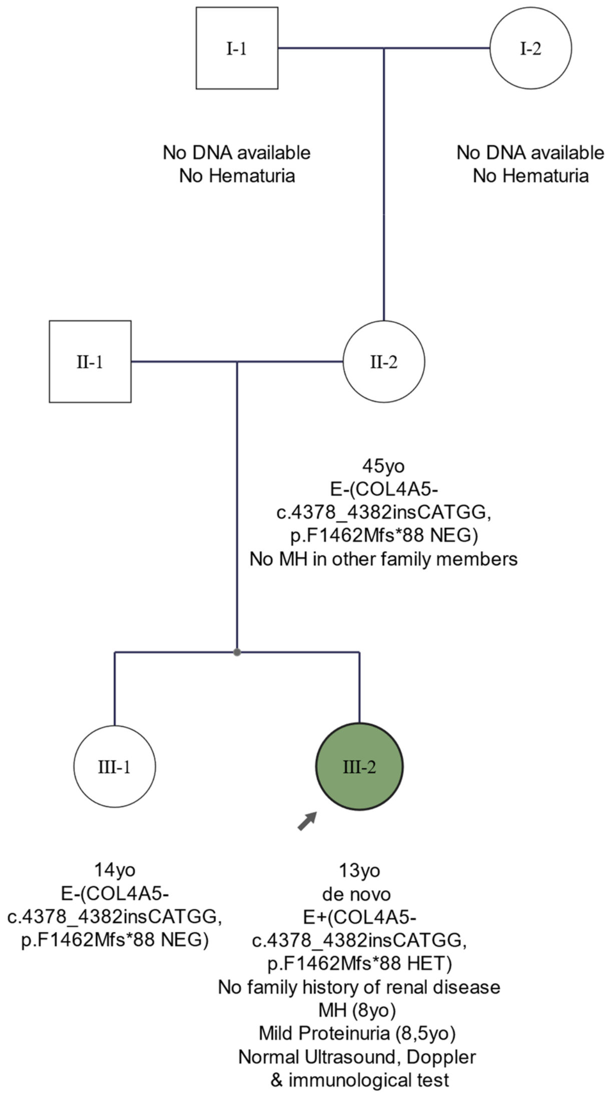

S1.13 Family GR1.13

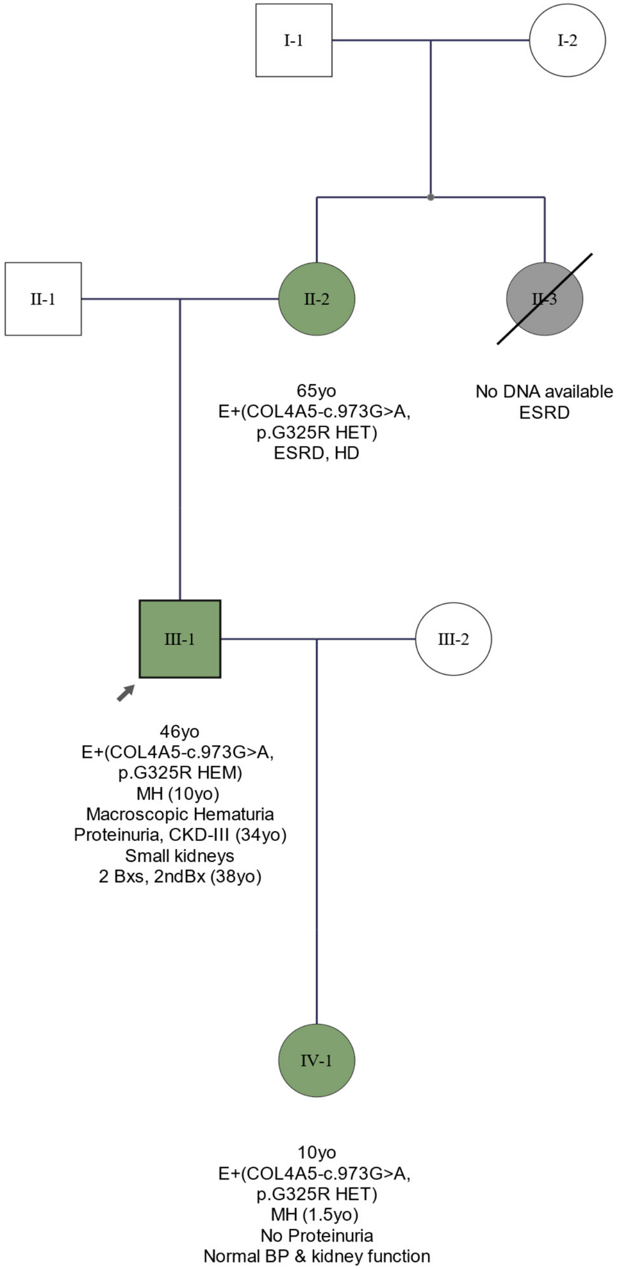

S1.14 Family GR1.14

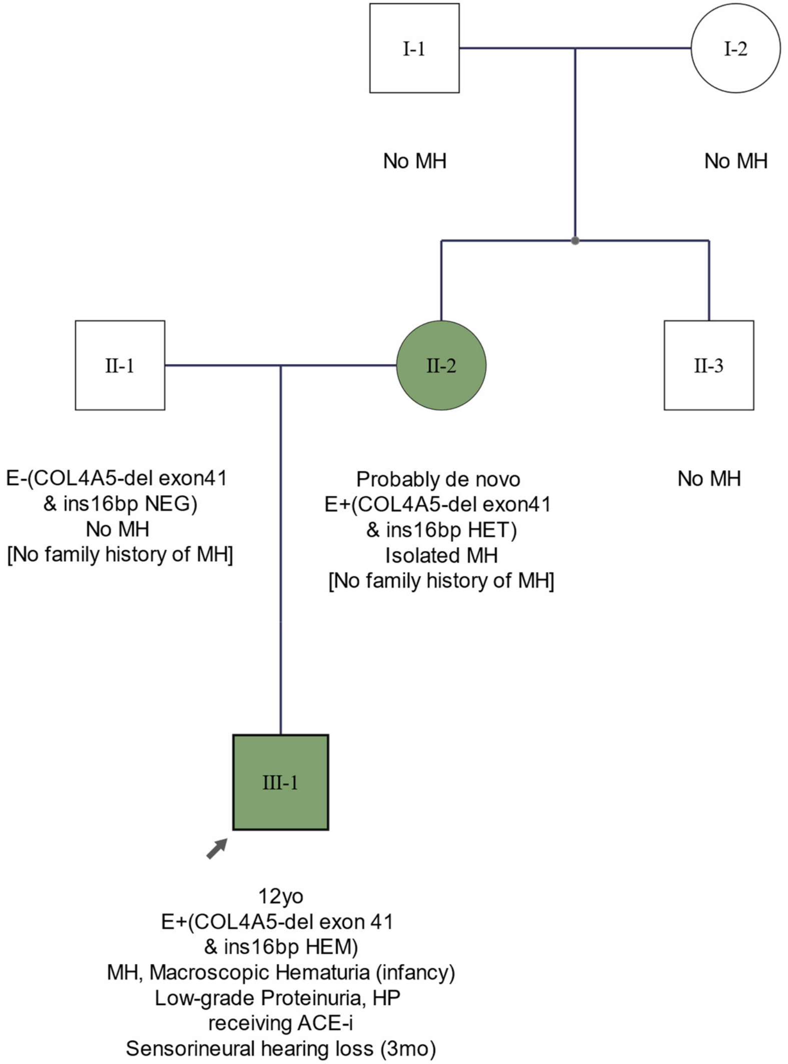

S1.15 Family GR1.15

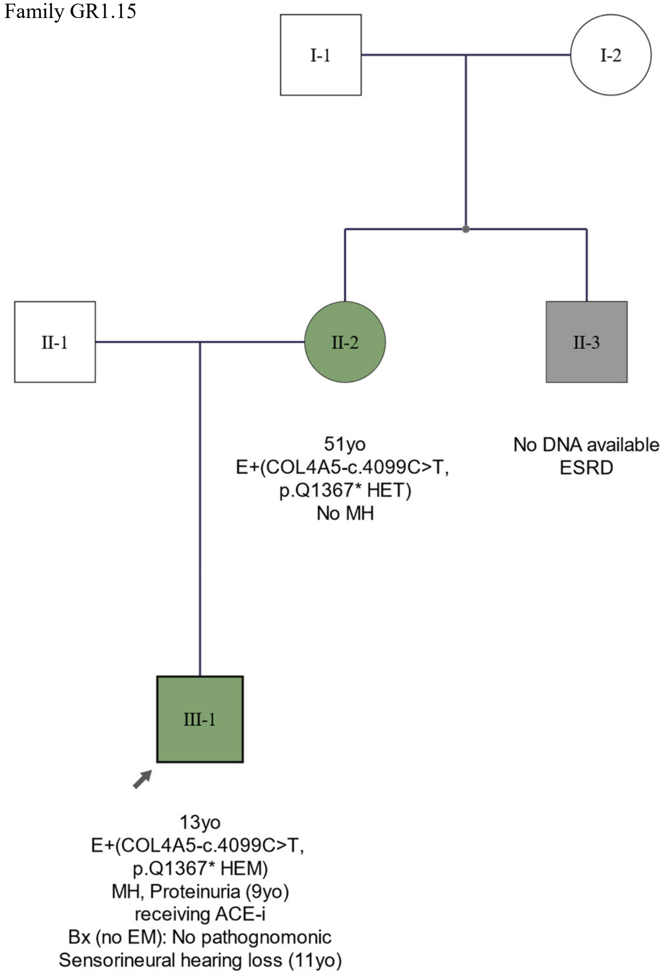

S1.16 Family GR1.16

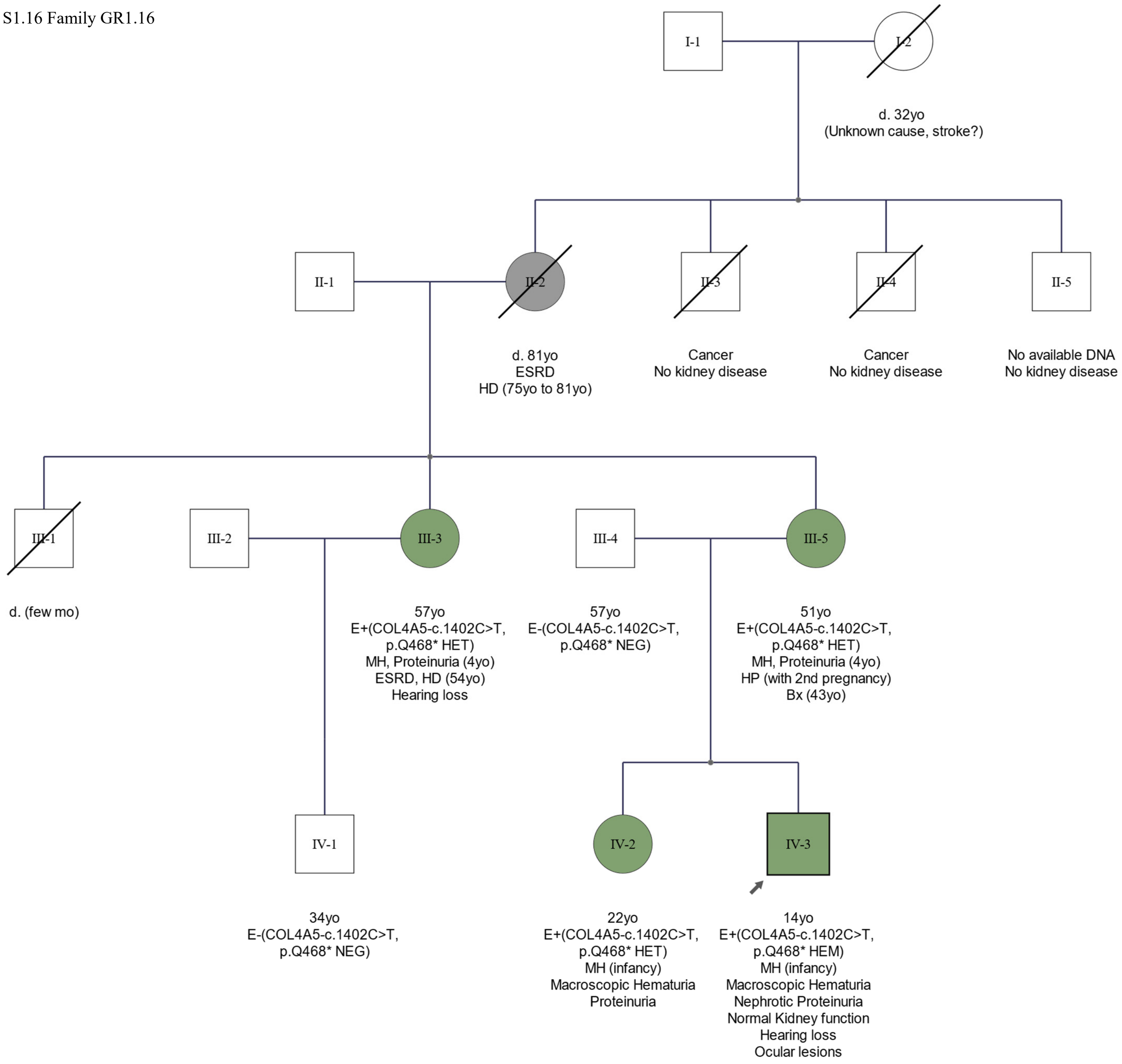

S1.17 Family GR1.17

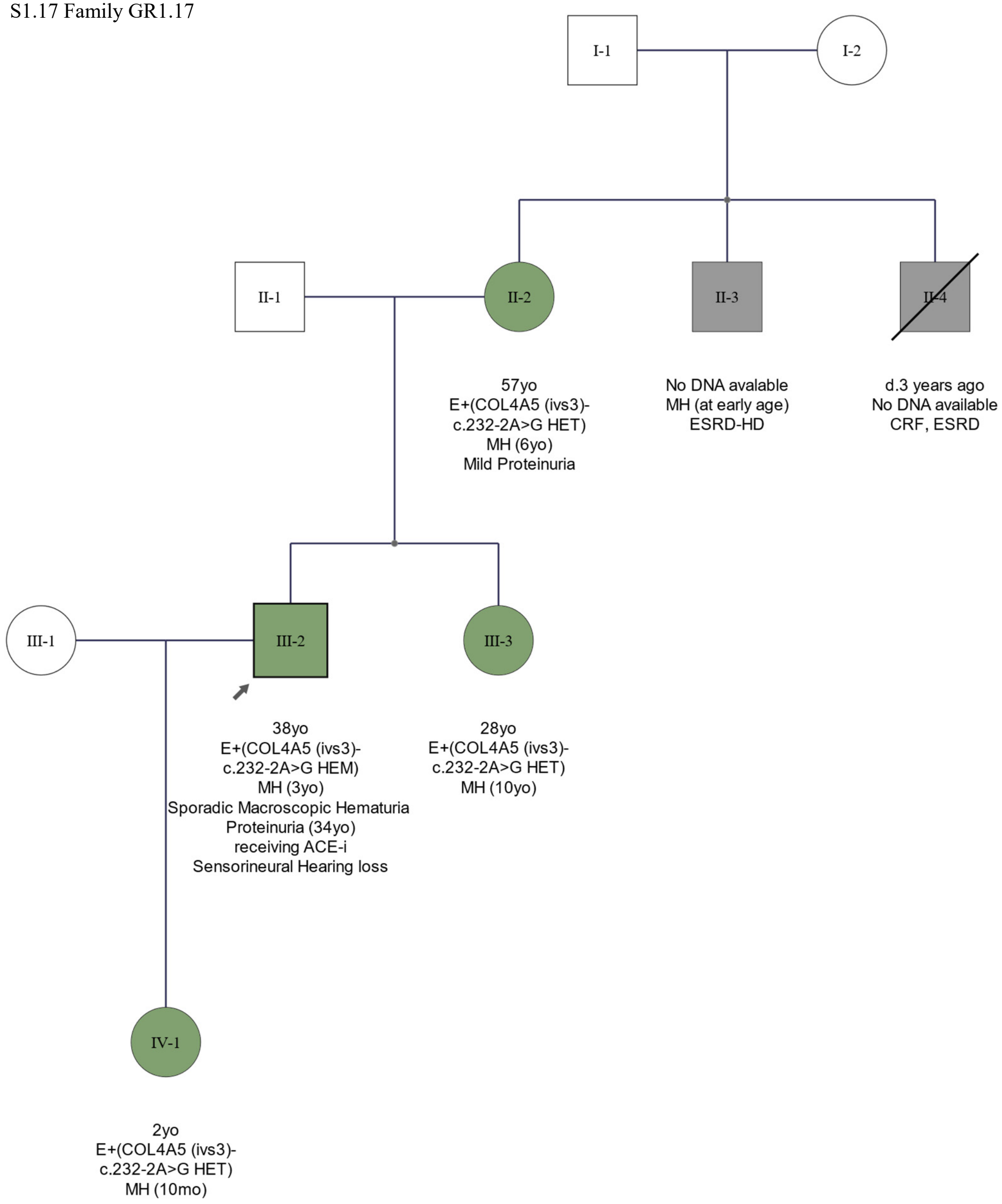

S1.18 Family GR1.18

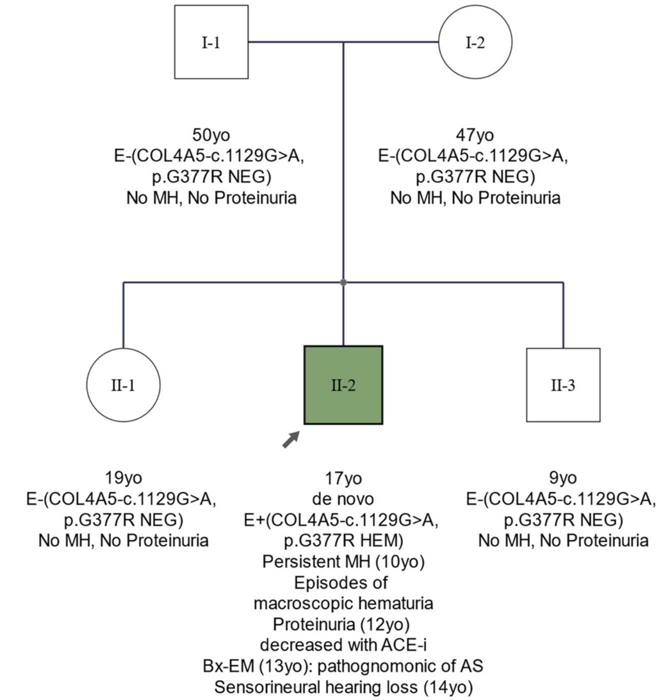

S1.19 Family GR1.19

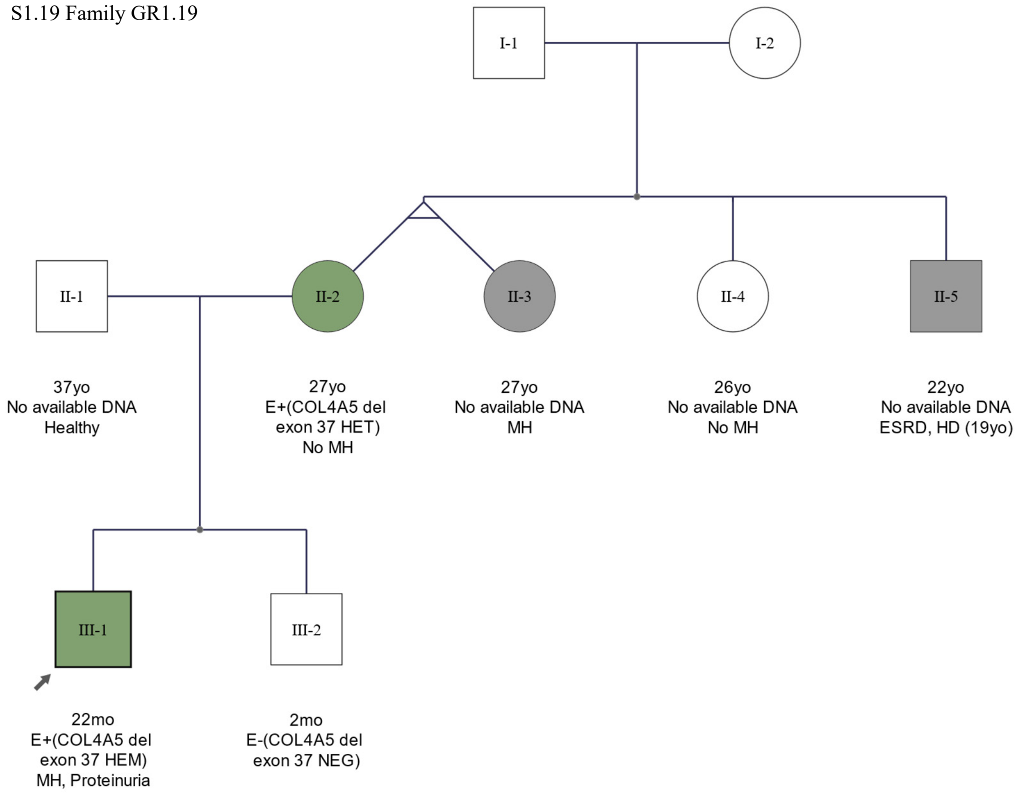

S1.20 Family GR1.20

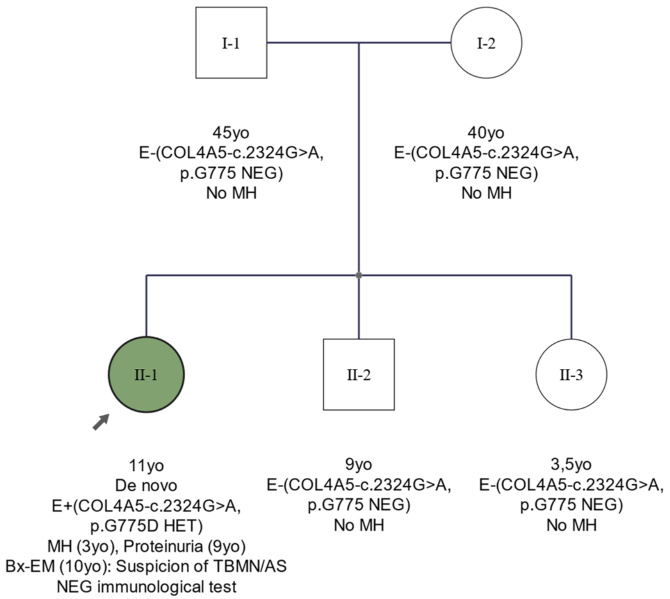

S1.21 Family GR1.21

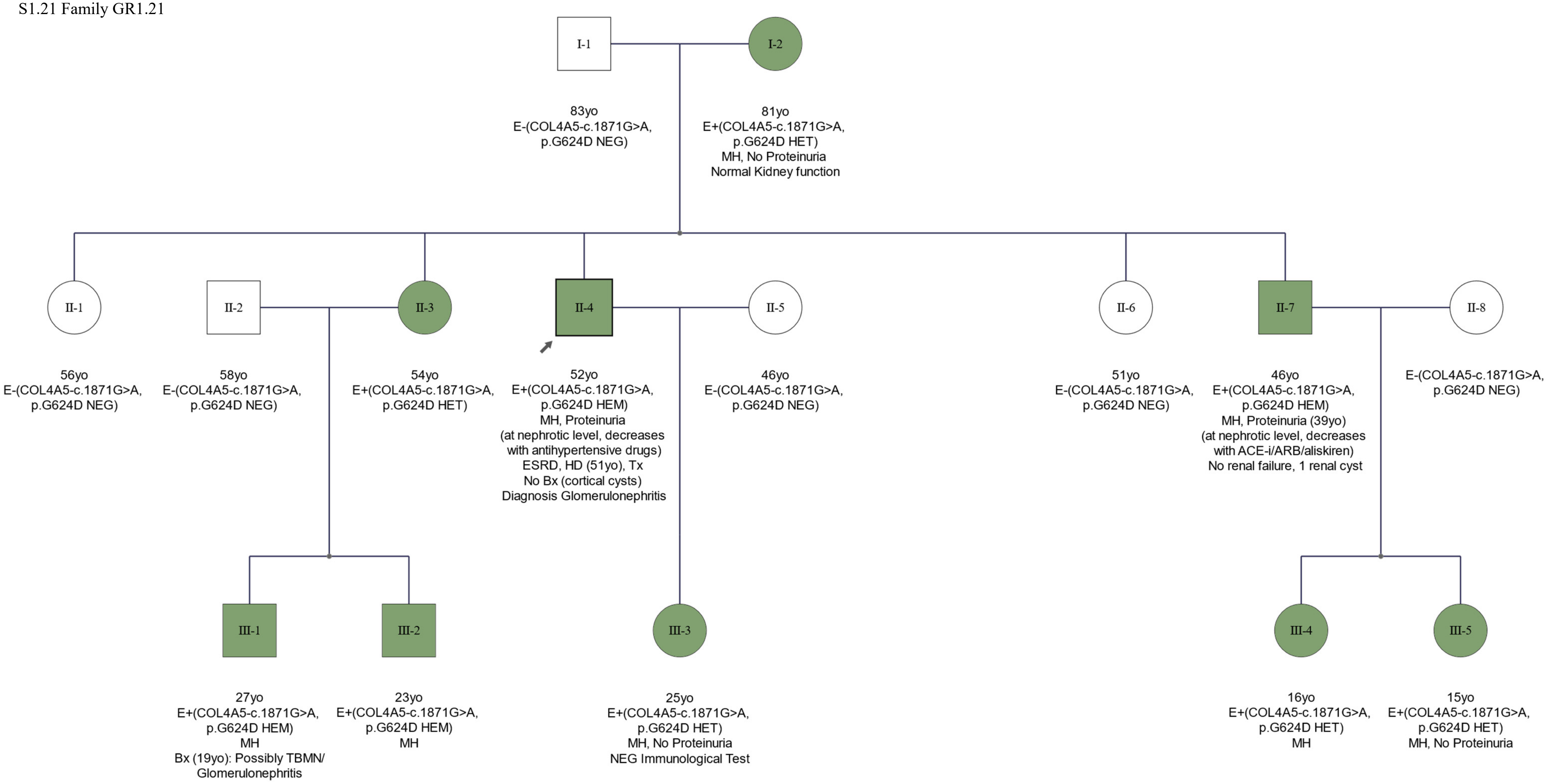

S1.22 Family GR1.22

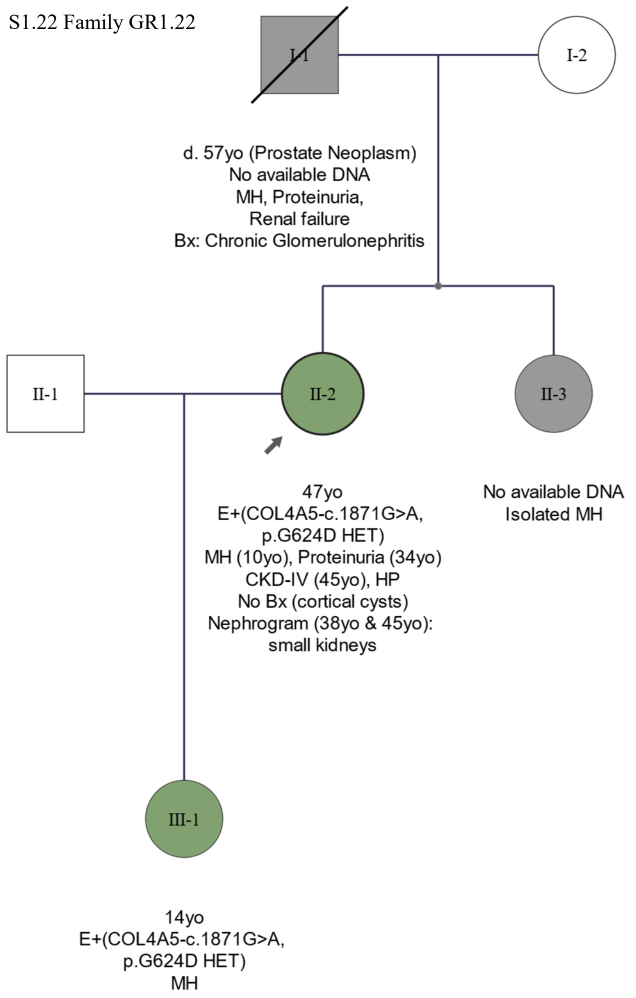

S1.23 Family GR1.23

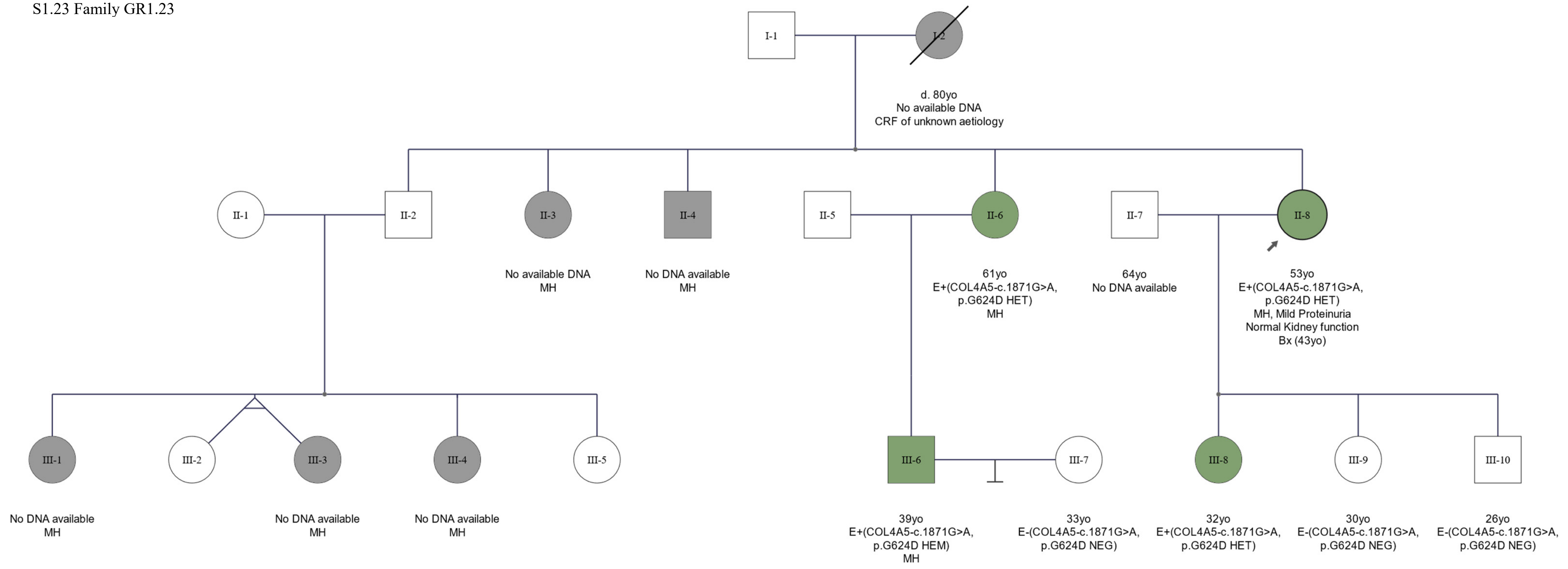

S1.24 Family GR1.24

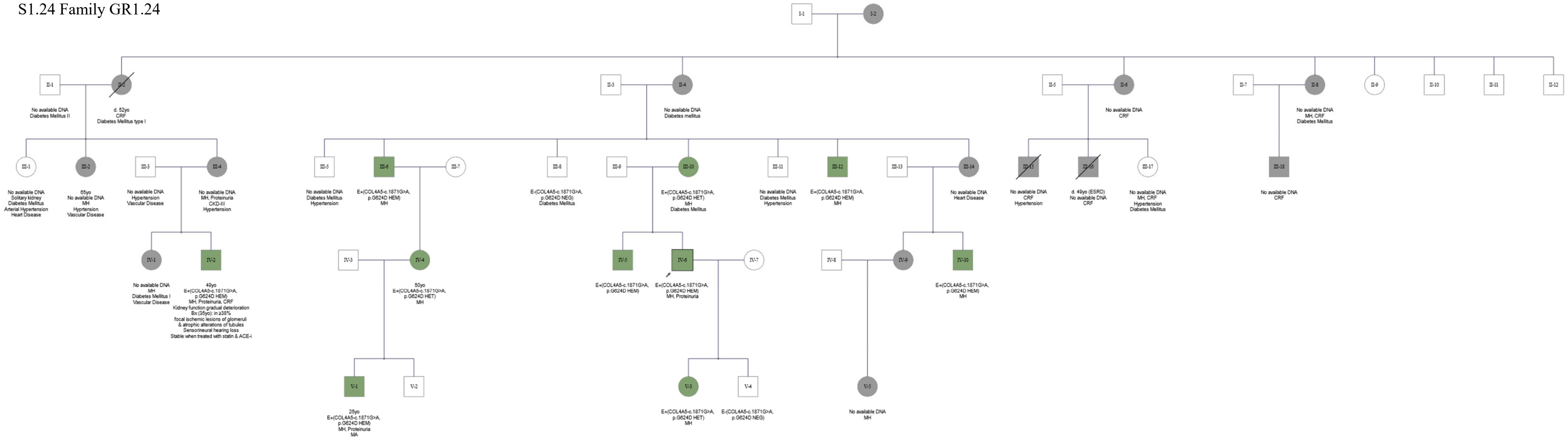

S1.25 Family GR1.25

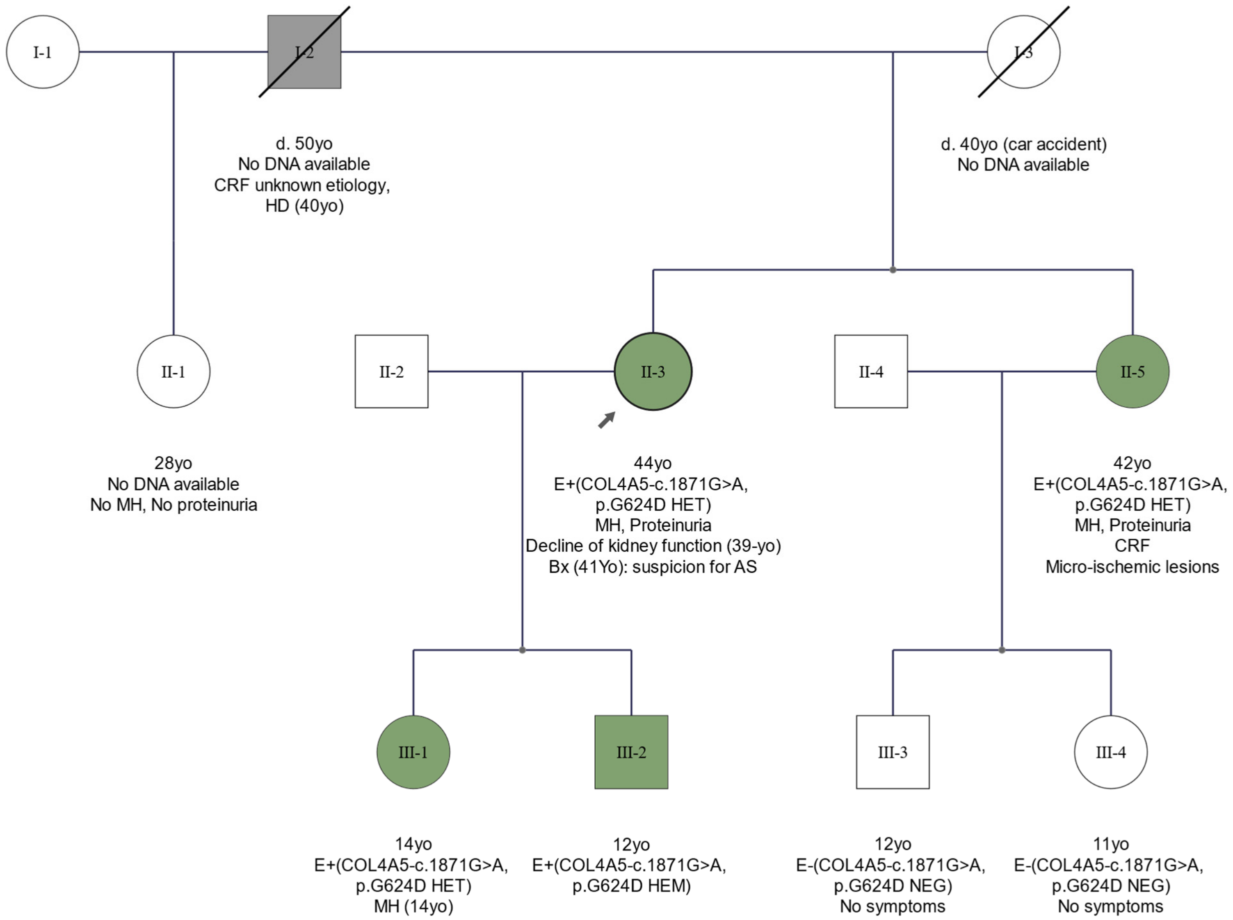

S1.26 Family GR1.26

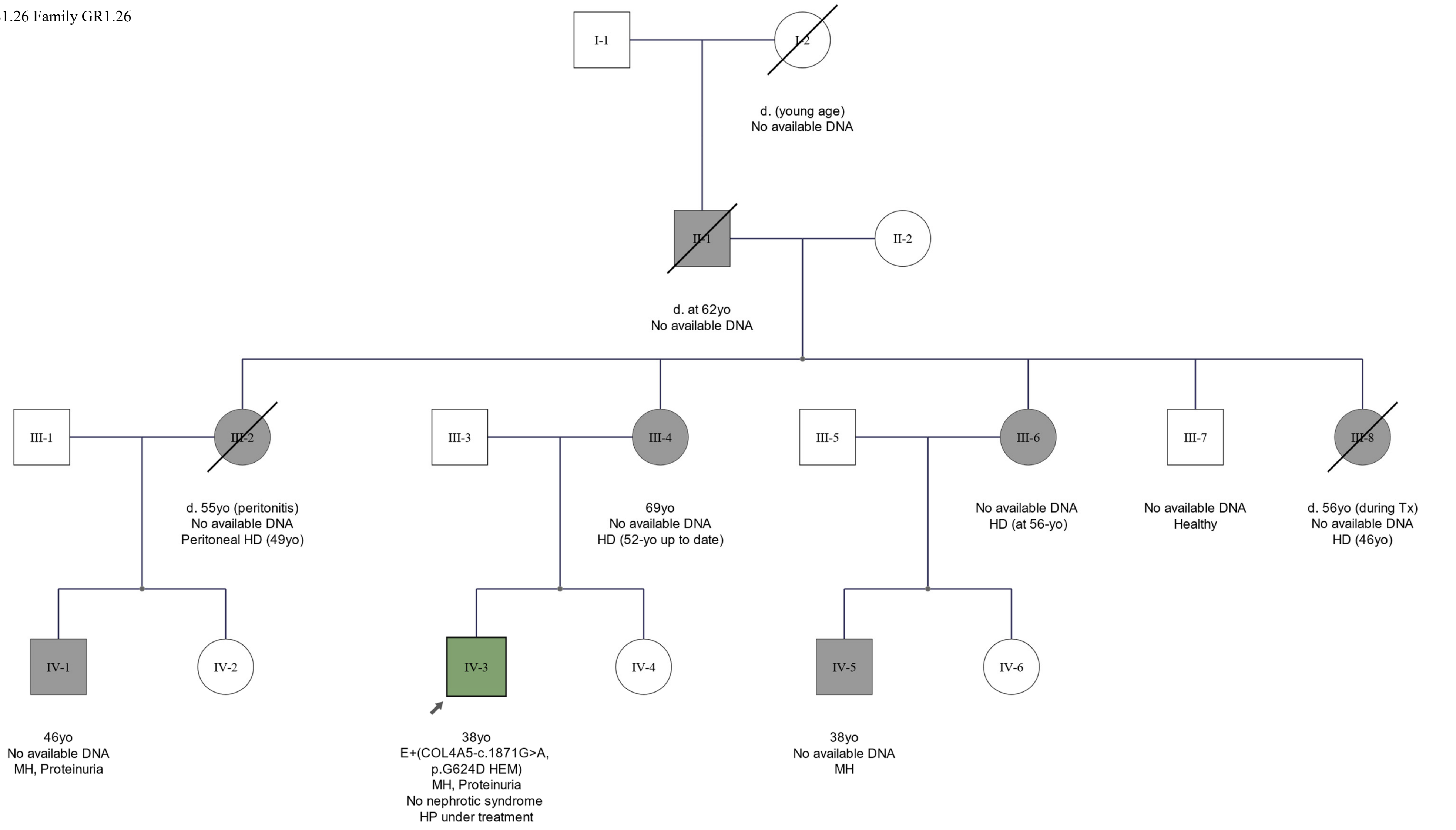

S1.27 Family GR1.27*

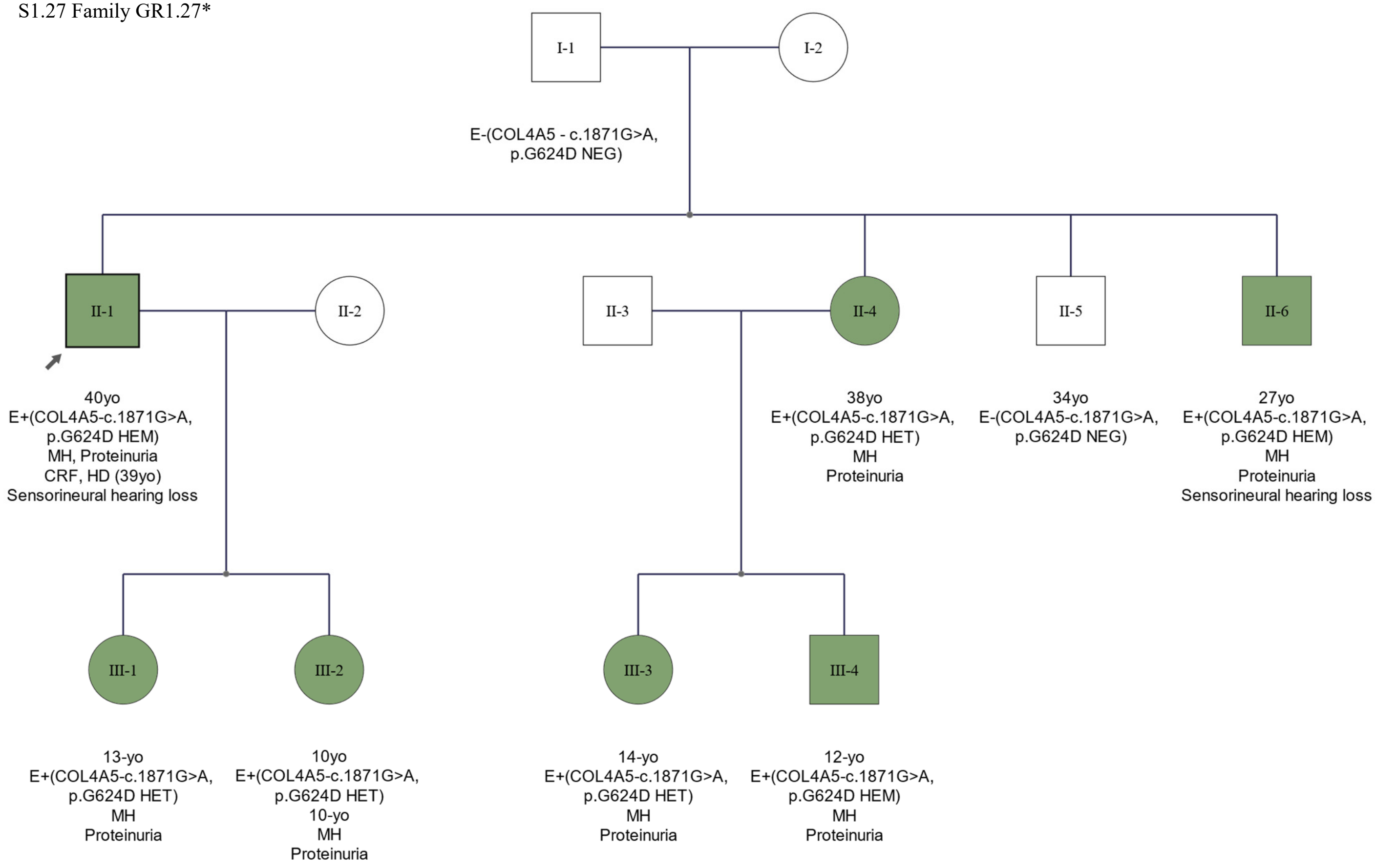

S1.28 Family GR1.28*

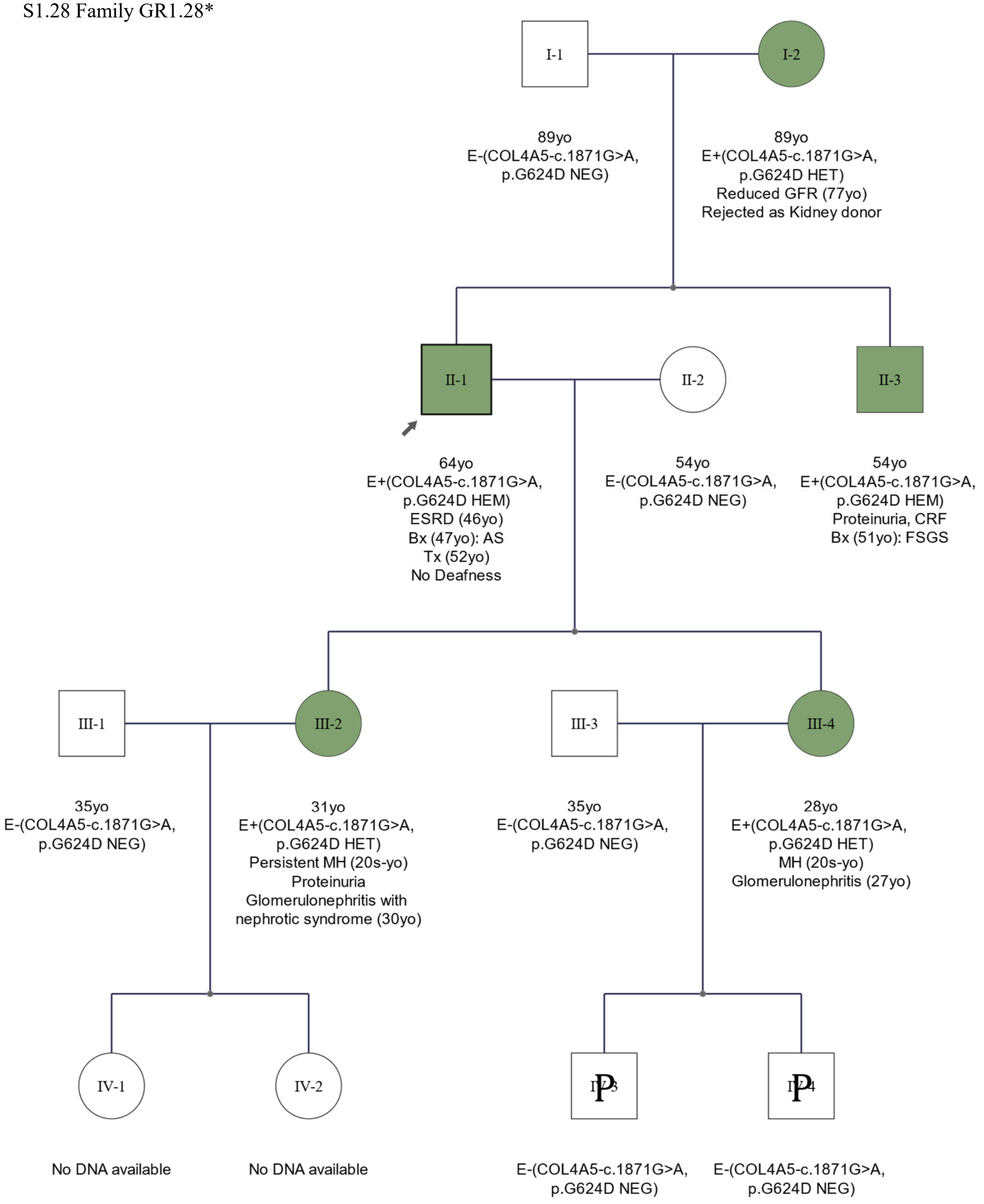

S1.29 Family GR1.29*

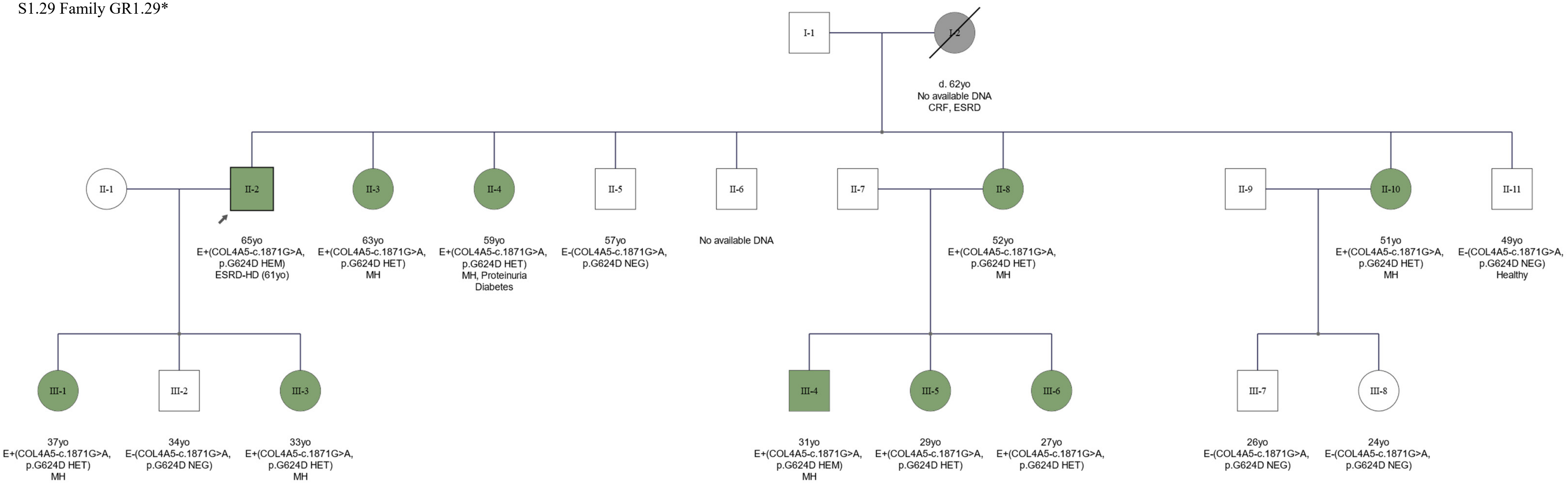

Figure S1 (S1.1-S1.29). Pedigrees of the 29 families (CR1.1, GR1.2-GR1.26, GR1.27*-GR1.29*) with a pathogenic variant in the X-linked *COL4A5* gene described in this study. The pedigrees of the nine families (six studied here and three mentioned in previous publications, indicated with an asterisk*) with the founder pathogenic variant *COL4A5*-c.1871G>A, p.G624D, are depicted in the end. We included mostly subjects we had DNA available for the diagnosis plus some healthy subjects to provide better overall pattern of inheritance. Patients filled in light green have been confirmed molecularly. Patients with symptoms but with no available DNA or / and are obligate carriers are represented with grey filled symbols in the pedigrees. Each subject is represented by a pedigree ID number, which includes a roman numeral for the generation and an Arabic number, a genotype if it is molecularly confirmed and some distinct clinical data. To manage family and clinical data in order to create the above pedigrees we used PhenoTips (REF https://pubmed.ncbi.nlm.nih.gov/23636887/) that uses "Open-Pedigree", an open-source tool, as its pedigree editor. Abbreviations: d.; died; yo, years old; mo, months; wk, weeks; E+, positive evaluation through genetic testing; E-, negative evaluation through genetic testing; HET, heterozygous; HEM, hemizygous; NEG, negative; MH, Microscopic hematuria; GFR, Glomerular filtration rate; CRF, Chronic Renal Failure; ESRD, End-stage renal disease; HD, hemodialysis; Bx, Biopsy; LM, Light Microscope; EM, Electron Microscope; FSGS, Focal segmental glomerulosclerosis; GBM, Glomerular basement membrane; TBMN, Thin basement membrane nephropathy; HP, hypertension; AS, Alport Syndrome; Tx, Transplanted;: GR, Greek origin; CR, Greek Cretan origin; ?, no data.
