# Supplementary material for: Novel and Founder Pathogenic Variants in X-Linked Alport Syndrome Families in Greece
Source: Genes (Basel). 2022 Nov 24;13(12):2203. doi: 10.3390/genes13122203 (PMC9778032; doi:10.3390/genes13122203)
Supplement: Supplementary file 1 [file genes-13-02203-s001.zip › Table S1_Hadjipanagi D et al.pdf]

Table S1. Information on the experimental conditions and reagents used for the verification by PCR and Sanger DNA sequencing of the 17 of 21 mutations discovered through NGS in this study. Underlined are 8 novel point mutations identified here. If no restriction enzyme was available for the examination of additional samples, the identification of the mutation was performed by direct Sanger DNA sequencing. Four other structural mutations, involving deletions, reported in this work were identified with the use of MLPA analysis.

Gene	Exon	Coding	Protein	Forward primer sequence	Reverse primer sequence	Tm (^{0}C)	PCR product (bp)	Restriction enzyme	Cleavage products (bp)
COL4A5	3	c.232-2A>G	NA	5'-GATATAACTTCCAGACGGTTTGTGT-3'	5'-AACAGCATCTTCCAAAAGCATC-3'	60	428	NA	NA
COL4A5	7	c.438+5G>A	NA	5'-GTAATTGGAAAGTGAAGGCTAATGA-3'	5'-ACACCTGCACAACTTCTAGAAACAT-3'	64	700	NA	NA
COL4A5	17	c.973G>A	p.G325R	5'-AGCTCTGAGTTCTCACCAAGATTC-3'	5'-GGACTGTGATTTATGGCAATGG-3'	65	470	NA	NA
COL4A5	19	c.1129G>A	p.G377R	5'-AGTTGGAGGATGGGAGTGATAC-3'	5'-AAAGGACTTGTGTCTCCTACTTCC-3'	65	395	NA	NA
COL4A5	21	c.1402C>T	p.Q468*	5'-GGGTTTTGGTAGTTCTTGGATG-3'	5'-GTAAGCTAGAAAGAAGAGAGCGATCC-3'	66	481	NA	NA
COL4A5	25	c.1871G>A	p.G624D	5'-TGTTGGAGGAATGAGTAAGTG GAATGATCACACATACCATC-3'	5'-TTGTATGCCTTTTTCACCTAC TGGGCCTGGAGGGCCGATA-3'	66	308	EcoRV	269+39 (mutant)
COL4A5	26	c.2006G>A	p.G669D	5'-TTTACCATTGATTTACTCTTGC-3'	5'-AGTTACTTTGAAATAAATTCCTC-3'	52	150	BstEII	85+65 (normal)
COL4A5	29	c.2324G>A	p.G775D	5'-TTACTAAACCCTGTTTCCAATCCTT-3'	5'-AAAATGTGTAGCAGTAACAGAATGG-3'	60	481	NA	NA
COL4A5	30	c.2452_2454del ATA/ 2653insT	p.I818Wfs*36	5'-ATAGATTCCCTATCCTTTGCTCTTG-3'	5'-CTGTGGCTTGTACTTATCCACTTTT-3'	64	430	NA	NA
COL4A5	31	c.2510-2A>G	NA	5'-GAGAAGCACTGTCTTAGAGCAATCTG-3'	5'-CTCATCCTTAAGGGATCACGTC-3'	64	488	NA	NA
COL4A5	32	c.2719C>T	p.P907S	5'-GGCCATCTTTAGAGATTAGCTACCAG-3'	5'-GGTATCCAAACTAAGCACGTAACC-3'	64	473	Hpy188III	246+227 (mutant)
COL4A5	32	c.2723G>A	p.G908E	5'-GGCCATCTTTAGAGATTAGCTACCAG-3'	5'-GGTATCCAAACTAAGCACGTAACC-3'	64	473	NA	NA
COL4A5	35	c.3044G>A	p.G1015E	5'-GTTGGGTTCTATCTGTGGACCTTA-3'	5'-AGAGAACACTACCCACTGATTACCTC-3'	64	378	NA	NA
COL4A5	41	c.3772G>A	p.G1258S	5'-GGTAGATTTGGGATTTGGTAGGAG-3'	5'-GATGGAGTTAGCTATGAACCAAGC-3'	60	512	NA	NA
COL4A5	45	c.4099C>T	p.Q1367*	5'-AATTTGTGTGTTTTGTCTCA-3'	5'-TAATAAAGATGATCTGCATTG-3'	52	177	NA	NA
COL4A5	47	c.4382_4383 insCATGG	p.F1462Mfs*88	5'-GTCTCCTAGCCCATGATATCTGAC-3'	5'-GAAGCCCCAAAAGAATAGAGAGAG-3'	65	483	NA	NA
COL4A5	47	c.4428C>G	p.C1476W	5'-GTCTCCTAGCCCATGATATCTGAC-3'	5'-GAAGCCCCAAAAGAATAGAGAGAG-3'	65	483	NA	NA
